# Spin pumping during the antiferromagnetic–ferromagnetic phase transition of iron–rhodium

**DOI:** 10.1038/s41467-019-14061-w

**Published:** 2020-01-14

**Authors:** Yuyan Wang, Martin M. Decker, Thomas N. G. Meier, Xianzhe Chen, Cheng Song, Tobias Grünbaum, Weisheng Zhao, Junying Zhang, Lin Chen, Christian H. Back

**Affiliations:** 1 Department of Physics, Beihang University, Beijing, China; 2 Department of Physics, Technical University of Munich, Garching, Germany; 3 Institute of Experimental and Applied Physics, University of Regensburg, Regensburg, Germany; 4 School of Materials Science and Engineering, Tsinghua University, Beijing, China; 5 Fert Beijing Research Institute, Beihang University, Beijing, China; 6 Nanosystems Initiative Munich (NIM), Munich, Germany

**Keywords:** Magnetic properties and materials, Phase transitions and critical phenomena, Spintronics

## Abstract

FeRh attracts intensive interest in antiferromagnetic (AFM) spintronics due to its first-order phase transition between the AFM and ferromagnetic (FM) phase, which is unique for exploring spin dynamics in coexisting phases. Here, we report lateral spin pumping by which angular momentum is transferred from FM domains into the AFM matrix during the phase transition of ultrathin FeRh films. In addition, FeRh is verified to be both an efficient spin generator and an efficient spin sink, by electrically probing vertical spin pumping from FM-FeRh into Pt and from Py into FeRh, respectively. A dramatic enhancement of damping related to AFM-FeRh is observed during the phase transition, which we prove to be dominated by lateral spin pumping across the FM/AFM interface. The discovery of lateral spin pumping provides insight into the spin dynamics of magnetic thin films with mixed-phases, and the significantly modulated damping advances its potential applications, such as ultrafast spintronics.

## Abstract (web summary)

Iron–rhodium is a promising material for antiferromagnetic (AFM) spintronics applications. Here, the authors demonstrate a strong enhancement of damping during the ferromagnetic (FM) to AFM phase transition caused by lateral spin pumping from FM domains to the AFM matrix.

## Introduction

The B2-ordered material FeRh undergoes a first-order phase transition from the antiferromagnetic (AFM) to the ferromagnetic (FM) phase in the vicinity of room temperature accompanied by a lattice expansion of about 1% (ref. ^1^). FeRh has stood out as a highly intriguing material for applications in heat-assisted magnetic recording^2,3^. Nowadays, with the rapid development of AFM spintronics aimed at low power and ultrafast logic devices^4–9^, FeRh attracts renewed interest as a unique AFM due to its particular physical properties during the phase transition. Benefiting from the ability to grow thin films of high quality, intensified functional devices based on the phase transition of FeRh have been proposed^10–15^. Fundamentally, a closer insight into spin-dependent transport in its AFM state and the related spin dynamics during the phase transition are crucial issues. Although X-ray magnetic circular dichroism (XMCD)^13,16–18^, ferromagnetic resonance (FMR)^19–21^, and time-resolved methods^13,14^ have been adopted to study magnetization dynamics, direct experimental observation of spin generation and detection in FeRh is highly desired.

AFM metals with large spin–orbit coupling (SOC) can act as spin current detectors via the inverse spin Hall effect (ISHE), where a detectable transverse charge current is converted from a pure spin current^22^, demonstrating that AFM metals act similarly as heavy metals such as Pt, Ta, or W^6–8^. Spin pumping from FM across/into AFM has been reported^23–26^, where enhanced spin pumping efficiency around the AFM magnetic phase transition temperature *T*_Néel_ has been observed^24^. In comparison with FM/AFM heterostructures with explicit vertical spin pumping, FeRh is unique concerning its magnetic structure, since AFM and FM matrixes/domains embed in the bulk during the first-order phase transition^3,17,27^, making it an ideal candidate to investigate spin dynamics at the interface of FM and AFM domains in a single material. In this intriguing case, a landscape of lateral spin pumping accompanied by the transfer of angular momentum from FM domains into AFM surroundings is presented. In addition to the previously reported non-equilibrium non-local transfer of angular momentum by optically excited electrons within the ferrimagnetic GdFeCo alloy^28^, lateral spin pumping within the equilibrium magnetization precession regime^29^, e.g., excited by FMR, has remained unexplored.

In this work, besides vertical spin pumping from FeRh into the adjacent normal metal (Pt) which is detected by ISHE voltages, we mainly report the experimental observation of lateral spin pumping from FM- into AFM-FeRh across its phase transition, evidenced by an enhanced linewidth and damping. The crucial role of AFM-FeRh as a spin detector is further confirmed by vertical spin pumping experiments from Py into FeRh. In addition to the low damping of ~0.0023 in the FM state which makes it competitive for magnonic and spin-orbitronic applications^30^, the greatly enhanced damping related to the AFM phase during the phase transition opens prospects towards ultrafast logic devices. The observation of lateral spin pumping and modulated damping are fundamentally significant for understanding the spin dynamics in FeRh and might advance its practical application in AFM spintronics.

## Results

### Magnetization properties of FeRh during phase transition

**Fig. 1:**
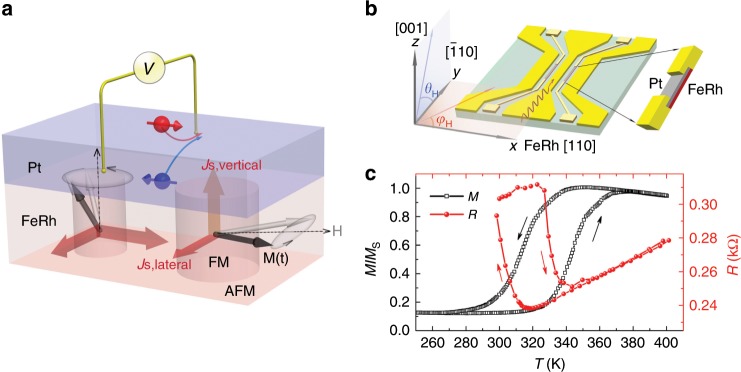
Illustration of spin pumping and the magnetic phase transition of FeRh. **a** Illustration of vertical spin pumping by magnetization dynamics from FM-FeRh into Pt, and lateral spin pumping from FM-FeRh into AFM-FeRh during the phase transition. Due to the ultrathin thickness (10 nm) and relatively large in-plane domain size (the diameter ranges from hundreds of nm to μm) of FeRh, the FM domains can be simply treated as flat pillars and the magnetic easy axis lies in-plane. **b** Schematic of the measurement configuration for spin pumping. The FeRh/Pt bilayers were patterned into long stripes and integrated into the gap of a CPW. **c** Temperature-dependent normalized magnetization (black squares) and four-point resistance (red circles) of FeRh (10)/Pt.

The bilayers studied here consist of FeRh(*t*)/Pt and FeRh(*t*)/Al, where *t* = 5 or 10 nm. α′-FeRh thin films were grown epitaxially on MgO (001) substrates by magnetron sputtering^15,31^. As capping materials we use either a 4-nm-thick Pt layer with strong SOC or an Al layer with weak SOC. As shown in Fig. 1, bilayers are fabricated into 6 µm wide and 300 µm long stripes which are placed in the gap between the signal line and ground planes of a coplanar waveguide (CPW). This configuration leads to out-of-plane rf-field excitation of the FeRh stripes^32^. The devices are placed in an external magnetic field which can be rotated both in the in-plane (*φ*_H_) and out-of-plane (*θ*_H_) directions. To provide an overview of the magneto-structural transition in FeRh, Fig. 1c presents the magnetization of full films measured by SQUID and the four-point resistance of corresponding stripes (10 µm × 60 µm) etched out of the same films (see Supplementary Note 1 for FeRh/Al). The *T*-dependent magnetization loops clearly demonstrate the phase transition between AFM and FM-FeRh, where the transition temperatures locate between 300 and 380 K for different samples. Since the AFM and FM states of FeRh contribute differently to the transport properties, an abrupt change in resistance is observed during the phase transition^30,33^.

### Vertical spin pumping in FeRh/Pt across phase transition

**Fig. 2:**
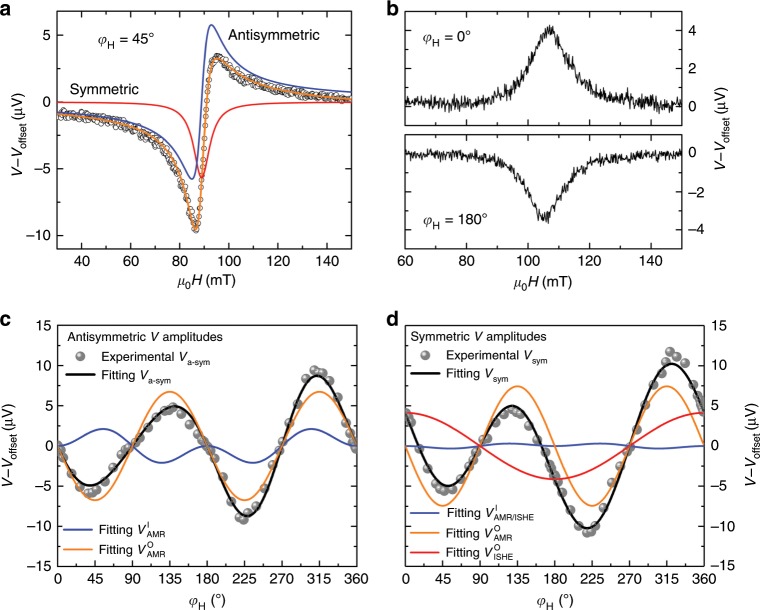
Angular dependence of d.c. voltages. **a** d.c. voltage signals obtained at *φ*_H_ = 45°, where fitting results include symmetric and antisymmetric parts. **b** Pure spin pumping voltage signals acquired at *φ*_H_ = 0° and 180°. **c**, **d** Antisymmetric and symmetric voltage signal amplitudes at FMR as a function of angle *φ*_H_. All data are recorded at 11 GHz and at 400 K for ferromagnetic FeRh (10)/Pt. The error bars in **c** and **d**, which are standard deviations obtained from the fit of the d.c. voltage spectra, are smaller than the size of the symbols.

In order to investigate the ISHE due to spin pumping and separate parasitic anisotropic magnetoresistance (AMR) from pure ISHE, spin pumping voltages as a function of in-plane rotation angle *φ*_H_ are studied. The device configuration with the stripe located in the gap of the CPW (out-of-plane excitation) has great advantages for easy separation of AMR and ISHE signals through analyzing the angle-dependent signals^32,34^. Note that finite inductive currents induced in the conductive Pt (Al) layers can lead to an additional in-plane magnetic driving field, which can be accurately determined by analyzing the dc voltages. A typical voltage trace (offset voltage *V*_offset_ is subtracted) for an excitation frequency of 11 GHz measured at 400 K and *φ*_H_ = 45° is shown in Fig. 2a. To fit the lineshape of the spectra, symmetric voltage components *V*_sym_*L*_sym_ (red line) and antisymmetric ones *V*_asym_*L*_asym_ (blue line) are introduced. The antisymmetric part is solely a consequence of AMR following *L*_asym_ = −4Δ*H*(*H*−*H*_R_)/[4(*H*−*H*_R_)^2^ + Δ*H*^2^], where *H*_R_ is the resonance field and Δ*H* is the linewidth (full-width at half-maximum, FWHM). In turn, the symmetric part contains both AMR and ISHE signals, and the lineshape can be described by *L*_sym_ = Δ*H*^2^/[4(*H*−*H*_R_)^2^ + Δ*H*^2^] (ref. ^34^). The antisymmetric and symmetric d.c. voltage amplitudes at FMR as a function of angle *φ*_H_ are shown in Fig. 2c, d, which can be fitted by (for a detailed derivation see Supplementary Note 2).

1 $$V_{{\mathrm{a}} {\mbox{-}} {\mathrm{sym}}} = V_{{\mathrm{AMR}}}^{\mathrm{I}} + V_{{\mathrm{AMR}}}^{\mathrm{O}} = C^{\mathrm{I}}\sin ^2\varphi \cos \varphi + C^{\mathrm{O}}\sin 2\varphi ,$$

2 $$V_{{\mathrm{sym}}} =\,\, V_{{\mathrm{AMR}}/{\mathrm{ISHE}}}^{\mathrm{I}} + V_{{\mathrm{AMR}}}^{\mathrm{O}} + V_{{\mathrm{ISHE}}}^{\mathrm{O}} = D^{\mathrm{I}}\sin ^2\varphi \cos \varphi \\ + D^{\mathrm{O}}\sin 2\varphi + E^{\mathrm{O}}\cos \varphi .$$

Here *C*^I^ (*C*^O^) is the coefficient of *V*_a-sym_ due to in-plane (out-of-plane) excitation, *D*^I^ (*D*^O^) the coefficient of *V*_sym_ due to in-plane (out-of-plane) excitation, and *E*^O^ the magnitude of *V*_ISHE_. Note that *φ* is the angle of the magnetization with respect to the axis of FeRh [110], which is assumed to be equal to *φ*_H_ due to the fact that the magnitude of in-plane magnetic anisotropic fields are much smaller than *H*_R_. From the fitting, sizeable *V*_ISHE_ can be obtained, which shows opposite polarities at *φ*_H_ = 0° and 180° (Fig. 2b). This clearly verifies spin pumping from FeRh into Pt. In contrast to FeRh/Pt, no ISHE signals are detected for FeRh capped with Al (Supplementary Note 2) due to the much smaller spin Hall angle of Al (ref. ^32^).

**Fig. 3:**
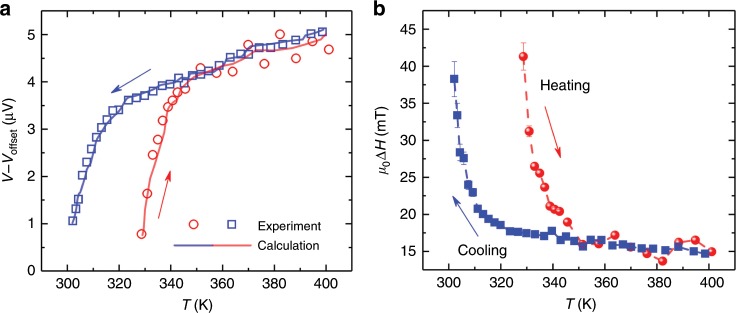
Vertical spin pumping voltages during the phase transition of FeRh. **a** ISHE voltages measured at *φ*_H_ = 0° during heating (red circles) and cooling (blue squares) for FeRh (10)/Pt at 11 GHz. The error bars are smaller than the size of the symbols. The solid lines are calculated results. **b** FMR linewidth as a function of *T*. The error bars are standard deviations obtained from the fit of the d.c. voltage loops.

We now turn towards vertical spin pumping during the AFM–FM phase transition. Figure 3a shows the ISHE voltages of FeRh (10)/Pt during heating and cooling at 11 GHz, *φ*_H_ = 0°. The voltages decrease continuously during the transition into the AFM state from 320 to 300 K. The traces of *V*_ISHE_ and linewidth (Fig. 3b) upon cooling and heating show an open window, which are consistent with the hysteretic magnetization/resistance traces shown in Fig. 1c. Similar tendencies have also been detected for thinner FeRh (5) films (see Supplementary Note 3). To have a better understanding of this phenomenon, a schematic diagram of the spin pumping scenario for FeRh/Pt during the phase transition is illustrated in Fig. 1a. Upon heating, FM domains nucleate in the AFM matrix and grow, until the whole sample is FM^17,27^. Conversely, cooling down from the FM state reverses the phase transition process and AFM domains nucleate in an FM matrix. It is known that, during the first-order phase transition of FeRh, the microscopic magnetization of separate FM-FeRh domains remain in the same magnetic state as the fully FM phase; it has been verified by XMCD that the orbital to spin moment ratio for Fe/Rh remains constant during the phase transition^16^. Thus, the reduction of the proportion of FM domains, together with the decreasing interfacial contact area between FM-FeRh pillars and the Pt film, will directly lead to a decrease of the vertically injected pure spin current and consequently the detectable voltages in Pt. Quantitatively, the measured data can be well reproduced by calculation results (solid lines in Fig. 3a) based on the dynamic magnetic susceptibility^33^, clarifying the process of the first-order phase transition in FeRh.

### Magnetic anisotropy

**Fig. 4:**
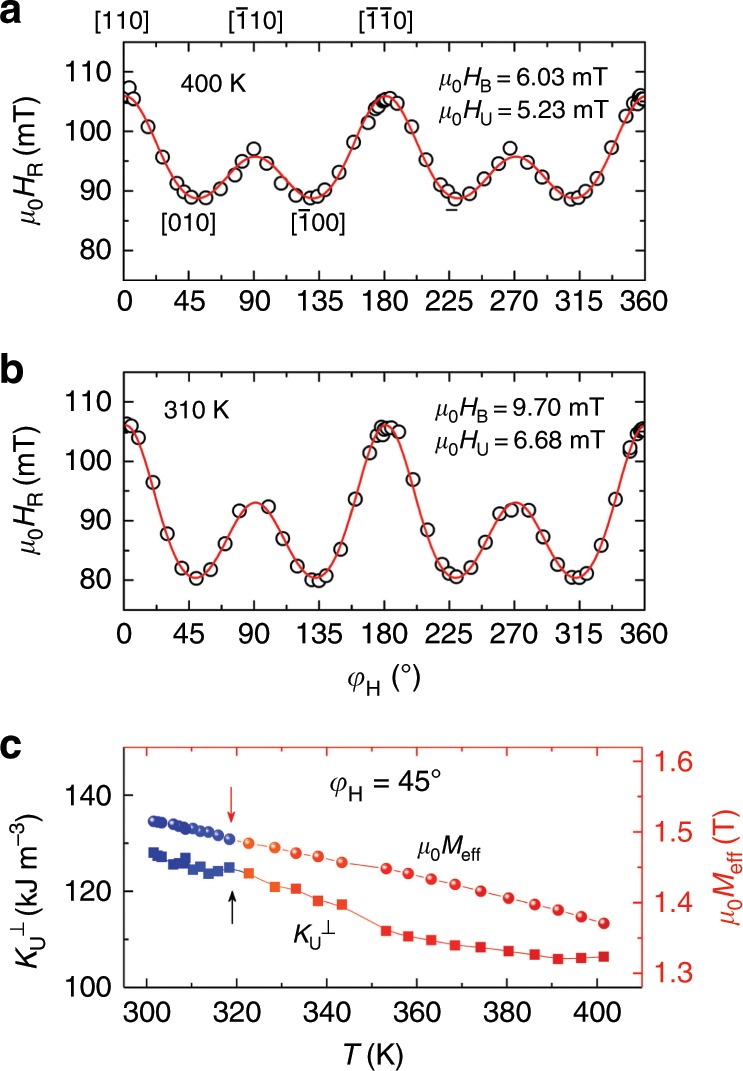
Magnetic anisotropies of FeRh/Pt at different temperatures. **a**, **b** In-plane rotation angle *φ*_H_-dependent resonance field of FeRh (10)/Pt at 11 GHz and at **a** 400 K and **b** 310 K. The data are fitted (solid lines) to extract the magnetic anisotropy parameters. **c** Temperature dependence of *μ*_0_*M*_eff_ (spheres) and $K_{\mathrm{U}}^ \bot$(squares) measured at *φ*_H_ = 45° of FeRh (10)/Pt during cooling into the phase transition region (blue spheres and squares). The error bars, which are standard deviations obtained from the fit, are smaller than the size of the symbols.

To explicate the change of magnetic susceptibility during the phase transition, which is the basis for exploring the spin dynamics of FeRh, we investigate the *T* dependence of the magnetic anisotropies. Figure 4a depicts the in-plane angular (*φ*_H_) dependence of the resonance field *μ*_0_*H*_R_ for FeRh (10)/Pt at 11 GHz and 400 K. The data can be well explained by considering the effective demagnetization field $\mu _0M_{{\mathrm{eff}}} = \mu _0M_{\mathrm{S}}-\mu _0H_ \bot = \mu _0M_{\mathrm{S}}-2K_{\mathrm{U}}^ \bot /M_{\mathrm{S}}$ of 1.37 T (with *H*⊥ the perpendicular magnetic anisotropy field and $K_{\mathrm{U}}^ \bot$ the corresponding uniaxial out-of-plane anisotropy energy constant), a cubic in-plane biaxial anisotropy field *μ*_0_*H*_B_ (along [100] or [010] directions) of 6.03 mT and a uniaxial anisotropy field *μ*_0_*H*_U_ (along [$\bar 1$10]) of 5.23 mT (see Supplementary Note 4). When *T* decreases to 310 K in the phase transition region (Fig. 4b), the values of *μ*_0_*M*_eff_, *μ*_0_*H*_B_, and *μ*_0_*H*_U_ increase due to the increase of magnetization. The *T* dependences of *μ*_0_*M*_eff_(*T*) and $K_{\mathrm{U}}^ \bot (T)$ obtained from the frequency-dependent *μ*_0_*H*_R_ along the in-plane easy axis (*φ*_H_ = 45°) are summarized in Fig. 4c. The increase of *μ*_0_*M*_eff_(*T*) during cooling is simply related to the increase of the saturation magnetization *μ*_0_*M*_S_(*T*) of FM-FeRh, which increases with decreasing temperature. Accordingly, the *T* dependence of $K_{\mathrm{U}}^ \bot (T)$ can be obtained from the experimental data for *μ*_0_*M*_eff_(*T*) in combination with *μ*_0_*M*_S_(*T*), which also exhibits an increase towards lower *T*. Below 320 K when FeRh goes into the phase transition region (as indicated by the arrows in Fig. 4c), no abrupt change of *μ*_0_*M*_eff_ and $K_{\mathrm{U}}^ \bot$ is observed, indicating again that the magnetic anisotropy of FeRh pillars remain, basically, unaffected during the phase transition. Similar behavior is observed for the heating up process (Supplementary Note 4).

### Detection of spin pumping voltages in FeRh

**Fig. 5:**
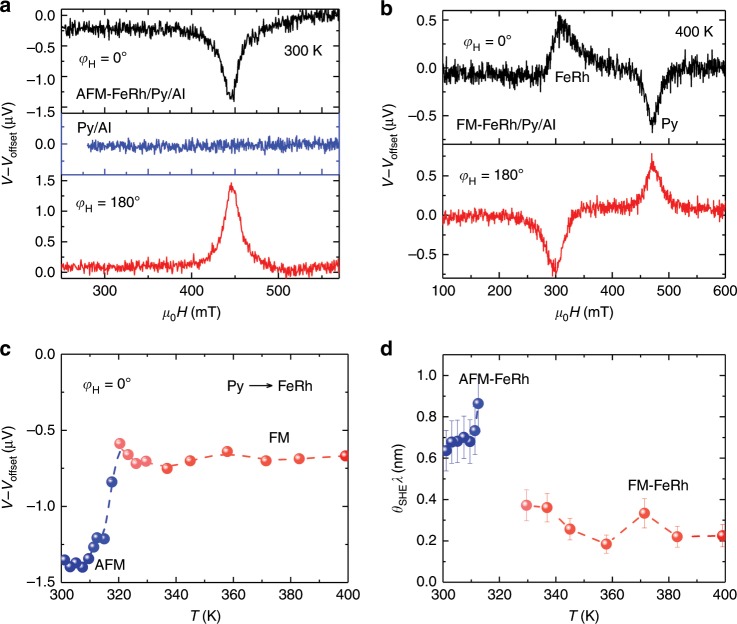
Vertical spin pumping in FeRh/Py/Al. **a** Pure spin pumping voltages acquired at *φ*_H_ = 0° and 180° at 22 GHz and 300 K, when FeRh is in AFM state. **b** Pure spin pumping voltages acquired at 400 K, when FeRh is in FM state. Mutual spin pumping from Py into FeRh (FMR of Py), or from FM-FeRh into Py (FMR of FeRh), is observed. **c** Temperature dependence of ISHE voltages measured at the FMR of Py. The error bars are smaller than the size of the symbols. **d** Temperature dependence of spin-to-charge conversion efficiency of FeRh, which shows a larger spin-to-charge conversion efficiency at the AFM phase, indicating that AFM-FeRh can be a good spin sink. The errors bars in **c** and **d** are standard deviations obtained from the fit.

To confirm the role of AFM-FeRh as an efficient spin sink and spin detector, we also investigate vertical spin pumping in an FeRh (10)/Py (15) bilayer capped with Al. FeRh in this bilayer also exhibits a reversible phase transition (Supplementary Note 5). As shown in Fig. 5a, b, clear *V*_ISHE_ features, i.e., symmetric spectra with opposite polarities at *φ*_H_ = 0°/180°, have been observed at 300 and 400 K. This unambiguously proves that the spin current is pumped from Py into FeRh, either into the FM or the AFM state, and transforms into a charge current due to the ISHE of FeRh. Moreover, a clear signal induced by spin pumping from FM-FeRh into Py has also been observed (400 K). Since this is not in the focus for the present discussion, we ignore it here. The crucial role of FeRh as a spin sink is further evidenced by the enhanced linewidth and damping compared to that of pure Py (see Supplementary Note 5). The spin Hall angle *θ*_SHE_ of FeRh can be quantified by

3 $$V_{{\mathrm{ISHE}}} = RI_{\mathrm{C}} = R\frac{{2{{e}}}}{\hbar }\theta _{{\mathrm{SHE}}}J_{\mathrm{S}}w\lambda \tanh \frac{d}{{2\lambda }},$$

where *R* is the resistance of the FeRh/Py bilayer, *I*_C_ the charge current induced by the ISHE, *e* the electronic charge, *ħ* the Dirac constant, *w* the width of the stripe, *λ* the spin diffusion length of FeRh, *d* the thickness of FeRh, and *J*_S_ the magnitude of spin current at the interface. Instead of *θ*_SHE_, we use the product of *θ*_SHE_ and *λ*, *θ*_SHE_*λ* in unit of nm, to determine the spin-to-charge efficiency^35^. This is based on the condition that *λ* of FeRh is assumed to be much smaller than *d*, which holds also for other AFMs^23^. Based on the temperature-dependent *V*_ISHE_ shown in Fig. 5c, the magnitude of *θ*_SHE_*λ* of FeRh as a function of temperature is plotted in Fig. 5d (for details see Supplementary Note 5). Note that the data of FeRh around 320 K have been excluded due to the scattering of the linewidth, probably related to inhomogeneities at the FeRh/Py interface. The magnitude of *θ*_SHE_*λ* is comparable to heavy metals^22^, due to the strong SOC arising from the 4*d* electrons of Rh in FeRh. A ~2 times larger *θ*_SHE_*λ* is found in the AFM phase, indicating that ΑFM-FeRh can be an efficient spin sink. This result, in turn, supports the crucial idea of the lateral spin pumping in single FeRh layer during its phase transition.

### Lateral spin pumping from FM-FeRh domains into AFM matrix

Apart from vertical spin pumping from FeRh into the Pt capping layer, lateral spin pumping from FM-FeRh into the AFM-FeRh surroundings during the phase transition can be expected. As illustrated in Fig. 1a, the side walls of the pillars provide channels for lateral spin pumping from the FM domains into the AFM matrix. Even though in this configuration injection of a spin current can be expected, it cannot be detected through a voltage across the FeRh/Pt layer: for the case of in-plane magnetization, a voltage due to the ISHE can only be expected in the out-of-plane direction^22,36^, which will average out due to the symmetric distribution of the injected spin current. However, a pronounced increase of linewidth is observed during the phase transition (Fig. 3b), indicating possible lateral spin pumping from FM-FeRh to AFM-FeRh.

It is known that both intrinsic and extrinsic effects can contribute to the *T* dependence of the linewidth. In addition to spin pumping, other extrinsic factors, including two-magnon scattering, inhomogeneity, and mosaicity broadening, could also enhance the experimental linewidth. Particularly along the in-plane easy axis (*φ*_H_ = 45°), mosaicity broadening is negligible and two-magnon scattering is minimized (see Supplementary Note 6). However, before we can conclude that the increase in *μ*_0_Δ*H* mainly arises from lateral spin pumping, other extrinsic effects contributing to the FMR linewidth must be excluded, which will be discussed below by analyzing the frequency dependence of *μ*_0_Δ*H*.

Through investigating the frequency-dependent linewidth of the spin pumping voltage spectra at different *T*, we are able to quantitatively study spin dynamics—here the effective damping—of FeRh during the phase transition. In the case of uniform magnetization, the FMR linewidth as a function of microwave frequency *f* for Gilbert damping (*α*) is given by^37,38^

4 $$\mu _0\Delta H = \mu _0{\mathrm{\Delta }}H_0 + 2\alpha \frac{{2{\uppi}f}}{\gamma }.$$

**Fig. 6:**
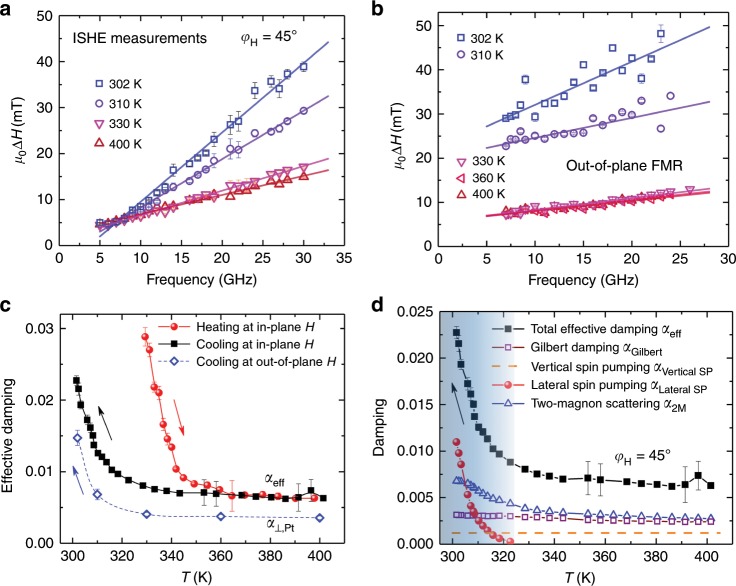
Temperature dependence of damping during the phase transition. **a**, **b** *f-*dependence of *μ*_0_Δ*H* at four typical temperatures for FeRh (10)/Pt. The values of *μ*_0_Δ*H* are extracted from **a** in-plane spin pumping measurements at *φ*_H_ = 45°, *θ*_H_ = 90°; **b** out-of-plane FMR measurements at *φ*_H_ = 45°, *θ*_H_ = 0°. The error bars are standard deviations obtained from the fit of the d.c. voltages and FMR spectra. **c** Effective damping at different *T* during heating and cooling. The solid spheres and squares are the data acquired from the spin pumping voltage measurements at *φ*_H_ = 45°, *θ*_H_ = 0°. The open rhombus represents the data acquired from out-of-plane (*φ*_H_ = 45°, *θ*_H_ = 90°) film-FMR measurements. **d** Four contributions to the total effective damping as a function of *T*: *α*_eff_(*T*) = *α*_Gilbert_(*T*) + *α*_Vertical SP_ (*T*) + *α*_Lateral SP_(*T*) + *α*_2M_(*T*). The blue shadow in **d** indicates the temperature region during the phase transition. The error bars of damping in **c** and **d** are obtained from the linear fitting of linewidth.

The damping calculated by linear fitting of the frequency-dependent *μ*_0_Δ*H* is called effective damping (*α*_eff_, since two-magnon scattering is included here^37,39^), which gives the upper limit for intrinsic damping. *μ*_0_Δ*H*_0_ represents the inhomogeneous term which is independent of *f* (also called zero frequency intercept). As plotted in Fig. 6a, linear fitting of *f* vs. *μ*_0_Δ*H* is adopted to allow a comparison between different *T*. Figure 6c summarizes the value of the calculated *α*_eff_ at different *T* during heating (solid spheres) and cooling (solid squares). It is interesting to note that *α*_eff_ is greatly enhanced during the phase transition, indicating a different dynamic behavior for FM/AFM domains coexisting in FeRh compared to the pure FM-FeRh. For all of the five samples with different thicknesses of FeRh and different capping metals, dramatic enhancement of *α*_eff_ is observed in the temperature region of the FM–AFM phase transition (see Supplementary Note 7).

To exclude the influence of two-magnon scattering which widely exists in FM films due to, e.g., misfit dislocations or defects, the out-of-plane FMR configuration is used where two-magnon scattering is switched off^37,39^. Since the spin pumping voltages cannot be detected when the external field is perpendicular to the interface, we conduct a control experiment by measuring standard full film-FMR. A wide stripe sized 18 µm × 400 µm directly beneath the signal line of the waveguide is measured. From linear fitting of the *f*-dependent *μ*_0_Δ*H* in the out-of-plane FMR measurements (*φ*_H_ = 45°, *θ*_H_ = 90°) at five typical temperatures (Fig. 6b), the calculated damping values *α*_⊥,Pt_(*T*) during cooling are compared to those obtained in the in-plane configuration in Fig. 6c. A larger value of *α*_eff_ is obtained for in-plane spin pumping compared with *α*_⊥,Pt_(*T*) for the out-of-plane FMR, and this difference is attributed to two-magnon scattering^37^. This is further confirmed by FMR measurements during out-of-plane rotation where *φ*_H_ is fixed to 45° (see Supplementary Note 8). The linewidth for the out-of-plane direction (*θ*_H_ = 90°) is smaller than that for the in-plane direction (*θ*_H_ = 0°), which clearly verifies the contribution of two-magnon scattering in the in-plane configuration^37–39^. It is worth emphasizing that by ruling out two-magnon scattering in the out-of-plane FMR measurements, *α*_⊥,Pt_(*T*) still increases from 0.0035 to 0.0147 by a factor of 4 (open rhombus in Fig. 6c) when cooling from the FM to the FM–AFM coexisting states. The enhanced damping indicates the experimental observation of lateral spin pumping, through careful separation of different contributions in the following discussion.

## Discussion

For FeRh/Pt bilayers, the total effective damping *α*_eff_(*T*) obtained from in-plane spin pumping measurements is composed of four parts:

5 $$\alpha _{{\mathrm{eff}}}\left( T \right) = \alpha _{{\mathrm{Gilbert}}}\left( T \right) + \alpha _{{\mathrm{Vertical}}\;{\mathrm{SP}}}\left( T \right) + \alpha _{{\mathrm{Lateral}}\;{\mathrm{SP}}}\left( T \right) + \alpha _{{\mathrm{2M}}}\left( T \right),$$

where *α*_Gilbert_(*T*) is the intrinsic Gilbert damping, *α*_Vertical SP_(*T*) the vertical spin pumping from FeRh into Pt, *α*_Lateral SP_(*T*) the damping contribution by lateral spin pumping from FM-FeRh into AFM-FeRh, and *α*_2M_(*T*) the damping contribution by two-magnon scattering. In comparison, the obtained damping from out-of-plane FMR (*α*_⊥_(*T*), open rhombus in Fig. 6c) excludes *α*_2M_(*T*): *α*_⊥,Pt_(*T*) = *α*_Gilbert_(*T*) + *α*_Vertical SP_(*T*) + *α*_Lateral SP_(*T*), but also increases dramatically during the phase transition. For FeRh/Al at 400 K, *α*_⊥,Al_ (0.0023) is smaller than *α*_⊥,Pt_ (0.0035) due to the lack of vertical spin pumping (*α*_Vertical SP_). Thus the value of *α*_Vertical SP_ is estimated to be 0.0012 and is basically a constant during the whole temperature range (see Supplementary Note 8), as shown by the dashed line in Fig. 6d. This is as expected because the interface properties between FeRh and Pt remain essentially unchanged during the phase transition, in spite of the reduced contacting areas. The effective spin mixing conductance $g_{{\mathrm{eff}}}^{ \uparrow \downarrow }$ is estimated to be 9.20(±0.53) × 10^18^ m^–2^, which is comparable to the magnitude of other FM/heavy metal bilayer structures^29,32,38^.

To highlight the crucial role of lateral spin pumping for the enhancement of damping, the intrinsic Gilbert-like damping *α*_Gilbert_(*T*) as a function of *T* is discussed first. Based on the microscopic theory of Gilbert damping which originates from spin–orbit scattering of band electrons in FM metals, *α*_Gilbert_(*T*) is expected to be quantitatively related to the out-of-plane magnetic anisotropy, which is also a second-order effect of the spin–orbit interaction^40,41^. When the whole FeRh film is in the FM state where lateral spin pumping is absent, *α*_Gilbert_(*T* *=* 350 ~ 400 K) is experimentally obtained according to *α*_⊥,Pt_(*T*) = *α*_Gilbert_(*T*) + *α*_Vertical SP_(*T*). It scales linearly with the uniaxial out-of-plane anisotropy $K_{\mathrm{U}}^ \bot (T)$ (see Supplementary Note 4), as already demonstrated in many FM systems^41^. Then, *α*_Gilbert_(*T*) in the whole temperature range across the transition temperature can be extrapolated based on the experimentally determined $K_{\mathrm{U}}^ \bot (T)$ (Fig. 4c), as shown by the empty squares in Fig. 6d. As calculated, the intrinsic damping *α*_Gilbert_ increases from 0.0024 to 0.0031 by ~29.2%, in good agreement with the values reported previously^30^.

It is worth emphasizing that the slight increase of *α*_Gilbert_ is consistent with the experimental resistance change during the phase transition. For a common FM, the damping will increase towards high (low) temperature as a consequence of interband (intraband) scattering, which is called resistivity-like (conductivity-like) behavior^30^. Specifically, for the case of FeRh where the phase transition occurs above 300 K, interband scattering is expected to dominate and the Gilbert damping *α*_Gilbert_(*T*) is generally proportional to the electrical resistivity *ρ* (ref. ^30^). So *α*_Gilbert_(*T*) is expected to increase by 28.9% during the phase transition, which is consistent with the derived result based on $K_{\mathrm{U}}^ \bot$. In addition to the different resistance contributions from FM- and AFM-FeRh, the resistance change during the phase transition also reflects scattering at the FM/AFM interface and lattice expansion induced electron–phonon scattering. Consequently, compared to the total increase of more than 400% for *α*_⊥,Pt_(*T*) or *α*_eff_(*T*), the increase of *α*_Gilbert_(*T*) during the phase transition can be ignored. Thus the *T* dependence of Gilbert damping and electron–phonon scattering cannot explain the sharp increase of the effective damping during the phase transition.

Importantly, the *T*-dependent *α*_Lateral SP_(*T*) due to lateral spin pumping can be obtained by subtracting *α*_Gilbert_(*T*) and *α*_Vertical SP_(*T*) from *α*_⊥,Pt_(*T*), as plotted by the red spheres in Fig. 6d. From the sharp increase of *α*_Lateral SP_(*T*) below 320 K, it can be concluded that the lateral spin pumping plays a crucial role in enhancing the total effective damping. The lateral spin pumping in FM-FeRh/AFM-FeRh, where AFM-FeRh acts as a spin sink, is also supported by the electrically detected ISHE voltages in Py/FeRh bilayers caused by spin pumping from Py into AFM-FeRh (Fig. 5). In addition, we also demonstrate the contribution from two-magnon scattering, where *α*_2M_(*T*) is estimated by subtracting *α*_⊥,Pt_(*T*) from *α*_eff_(*T*), according to Eq. (5). As expected, *α*_2M_(*T*) (blue triangles in Fig. 6d) exhibits a clear increase during the phase transition, due to the increased inhomogeneity and scattering at the interface between FM and AFM domains. Although two-magnon scattering adds to the value of the total effective damping, it does not dominate compared with the dramatic enhancement of *α*_Lateral SP_(*T*) arising from lateral spin pumping. In analogy to FeRh/Pt, a dramatic increase of damping attributed to lateral spin pumping is also observed in FeRh/Al (see Supplementary Note 8), verifying that the detection of lateral spin pumping is independent of the capping material. In summary, during the phase transition of FeRh from FM into AFM, the FMR linewidth and effective damping are crucially enhanced, indicating distinct spin dynamics related to lateral spin pumping from the FM domains into the AFM surroundings, where the interface between FM and AFM-FeRh acts as the channel. In addition, other non-local contributions including two-magnon scattering, lattice expansion induced electron–phonon scattering, and interfacial exchange coupling are also verified to be not the dominant mechanisms for the greatly enhanced damping. Thus we conclude that lateral spin pumping is the main mechanism at work and significant angular momentum is transferred from the FM domains to AFM matrix. According to the greatly enhanced damping during the phase transition of FeRh and the high efficiency of spin sinking in AFM-FeRh, the lateral spin pumping between FM- and AFM-FeRh in this case could be more efficient than in traditional FM/NM bilayers. This study provides the scientific basis for understanding spin dynamics during a first-order phase transition.

## Methods

### Sample preparation

FeRh (*t* = 5 nm, 10 nm)/(Pt, Al) and FeRh (10)/Py (15)/Al are deposited on MgO (001) substrates by magnetron sputtering. α′-FeRh is grown at 300 °C and then annealed at 750 °C for 1.5 h. After cooling down to room temperature, either 4 nm Pt or 4 nm Al capping layers are deposited at a base pressure of 4 × 10^−7^ Pa.

### Device

The film stacks are patterned into wires of 6 µm width and 300 µm length and integrated into a CPW structure, using electron beam lithography combined with Ar ion milling and lift-off process. The CPW consists of a 50 µm wide signal line and 30 µm wide gap corresponding to an impedance of 50 Ω in the GHz frequency range. The bilayers are placed in the gap between the signal line and ground planes.

### Measurements

For spin pumping measurements, microwave currents with a frequency ranging from 6 to 30 GHz are used and the input microwave power is 25 dBm. The measurements are carried out in vacuum with a base pressure of 5 × 10^−5^ Pa and at varied temperatures from 300 to 400 K. For full film-FMR measurements, a modulation field of 1 mT together with lock-in amplification is used to increase the signal-to-noise ratio. Note that the four-point resistance measurement on the Hall bar and the spin pumping measurements are carried out simultaneously and on the same substrate to provide a precise temperature reference.

## Supplementary information

Supplementary Information

## Data Availability

The data that support the findings of this study are available from the corresponding author on request.
