# Supplementary material for: Spin pumping during the antiferromagnetic–ferromagnetic phase transition of iron–rhodium
Source: Nat Commun. 2020 Jan 14;11:275. doi: 10.1038/s41467-019-14061-w (PMC6959338; doi:10.1038/s41467-019-14061-w)
Supplement: Supplementary file 1 — Supplementary Information [file 41467_2019_14061_MOESM1_ESM.pdf]

Supplementary Information

Spin pumping during the antiferromagnetic-ferromagnetic phase transition of iron-rhodium

Yuyan Wang[1,2,3]*, Martin M. Decker[2,3], Thomas N.G. Meier[2,3], Xianzhe Chen[4], Cheng Song[4], Tobias Grünbaum[3], Weisheng Zhao[5], Junying Zhang[1], Lin Chen[2,3]*, Christian H. Back[2,3,6]

[1]Department of Physics, Beihang University, Beijing, China. [2]Department of Physics, Technical University of Munich, Garching b. Munich, Germany. [3]Institute of Experimental and Applied Physics, University of Regensburg, Regensburg, Germany. [4]School of Materials Science and Engineering, Tsinghua University, Beijing, China. [5]Fert Beijing Research Institute, Beihang University, Beijing, China. [6]Nanosystems Initiative Munich (NIM), Munich, Germany.

*Email: wangyy@buaa.edu.cn; lin0.chen@tum.de

Supplementary Note 1: Magnetization and transport properties of FeRh during phase transition

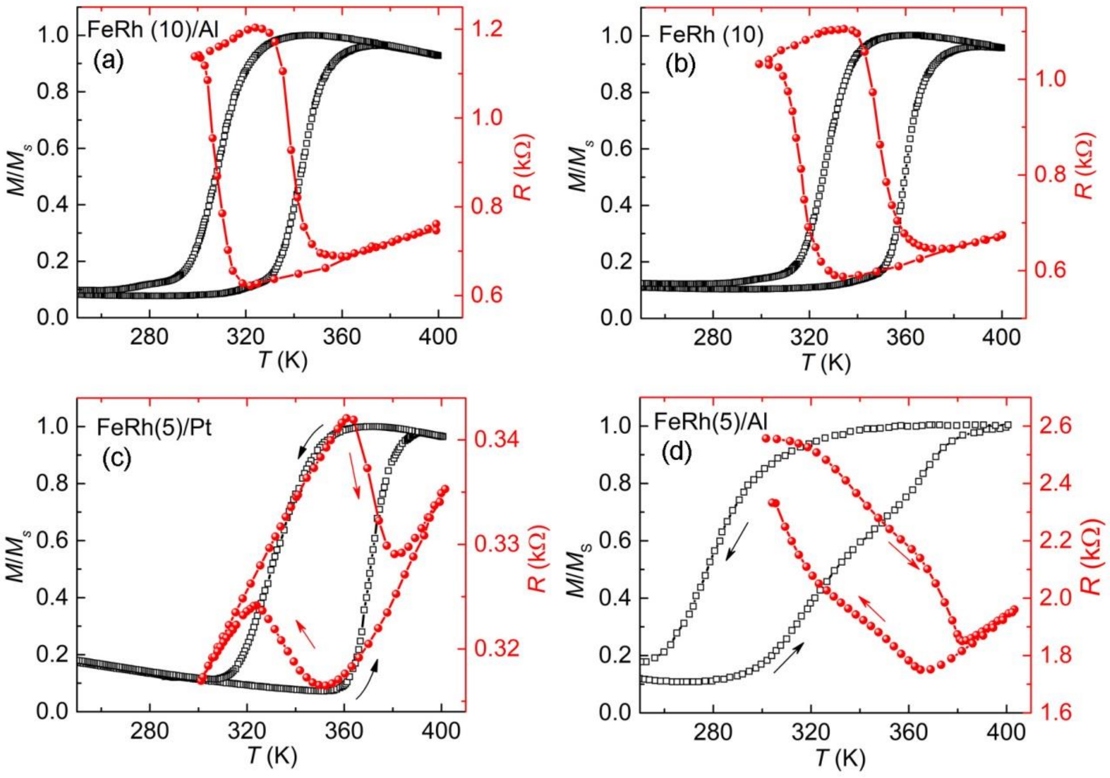

Supplementary Figure 1. Temperature dependent normalized magnetization (black squares) and four-point resistance (red circles) of (a) FeRh (10)/Al, (b) FeRh (10), (c) FeRh (5)/Pt, (d) FeRh (5)/Al.

The magnetization of the full films together with the four-point resistance of the stripes as a function of temperature are shown in Supplementary Figure 1. Compared with the sharp reversal of the loops for 10 nm-thick FeRh, the asymmetric as well as the broadened hysteresis shown in Supplementary Figure 1c and d indicate non-perfect α'-FeRh growth for the rather thin FeRh layers of 5 nm, where the strain generated by the epitaxial growth induces ferromagnetism and alters the transition in the vicinity of the substrate/film interface [1,2].

Supplementary Note 2: Angle dependent d.c. voltages in FeRh/Pt and FeRh/Al

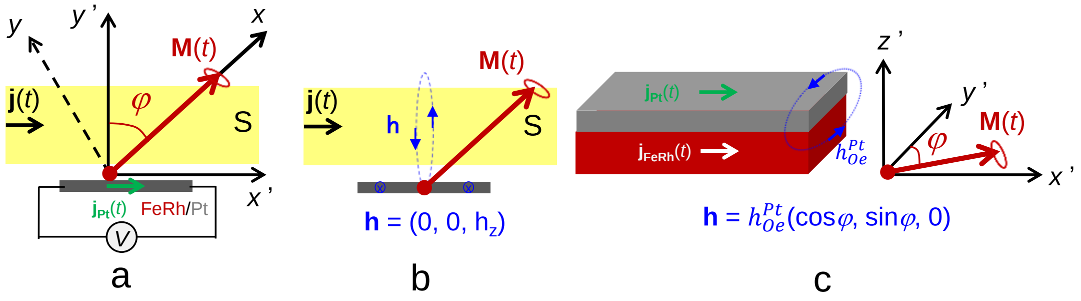

Supplementary Figure 2. (a) Schematic for the electrical detection of the inverse spin Hall effect induced by spin pumping in an FeRh/Pt bi-layer. For simplicity, the ground lines of the CPW are not shown. Two Cartesian coordinate systems (*x*', *y*', *z*') and (*x*, *y*, *z*) are used. The microwave current **j**(*t*) is along the *x*' direction, which generates an inductive current $\mathbf{j}_{\mathrm{Pt}}(t)$ and $\mathbf{j}_{\mathrm{FeRh}}(t)$ in Pt and FeRh in the same direction. The direction of the detected dc-voltage *V* is parallel to $\mathbf{j}_{\mathrm{FeRh}}$ (*t*), thus, a dc-voltage *V* originating from the anisotropic magneto-resistance effect of FeRh as well as from spin pumping should be considered. **M**(*t*) is the dynamic magnetization, **M**(*t*) = (M, m_y, m_z). φ is magnetization angle. In the present measurement configuration, two main excitations are considered. One is the out-of-plane excitation due to the Oersted field from the CPW as shown in (b). In the coordinate system of (*x*, *y*, *z*), the driving field can be written as **h** = (0, 0, h^{O}). The other one is the in-plane Oersted field $h_{\mathrm{Oe}}^{\mathrm{Pt}}$ due to the inductive current in Pt $\mathbf{j}_{\mathrm{Pt}}$ (*t*) as shown in (c). $h_{\mathrm{Oe}}^{\mathrm{Pt}}$ is along the *y*' direction, and in the coordinate system of (*x*, *y*, *z*), it can be written as **h** = $h_{\mathrm{Oe}}^{\mathrm{Pt}}$ ($\cos\varphi$, $\sin\varphi$, 0).

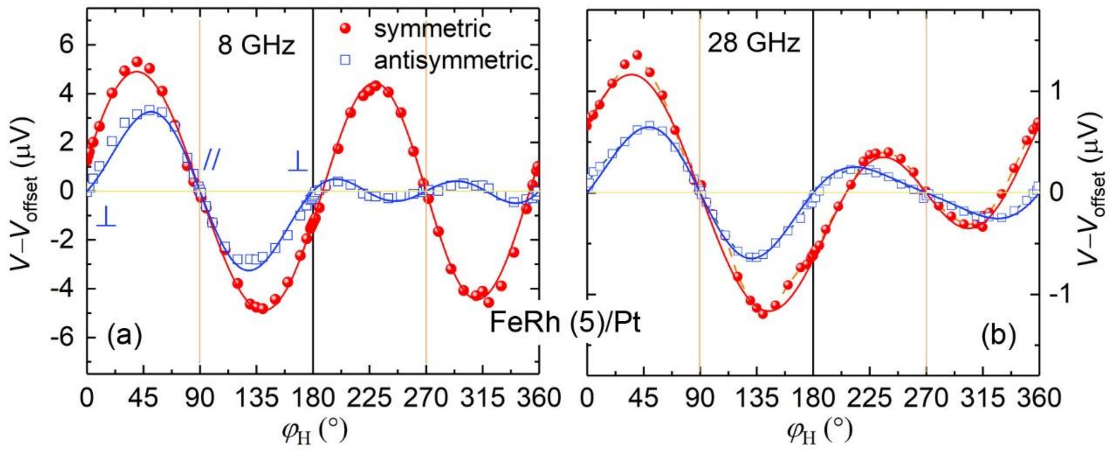

Supplementary Figure 3. Symmetric (red solid spheres) and antisymmetric (blue open squares) d.c. voltages as a function of angle φ_H for FeRh (5)/Pt at (a) 8 GHz, (b) 28 GHz. The red and blue lines are fitting results for the symmetric and antisymmetric components, respectively. The error bars, which are obtained from the fit of d.c. voltage loops as shown in Fig. 2a of the main text, are smaller than the size of the symbols.

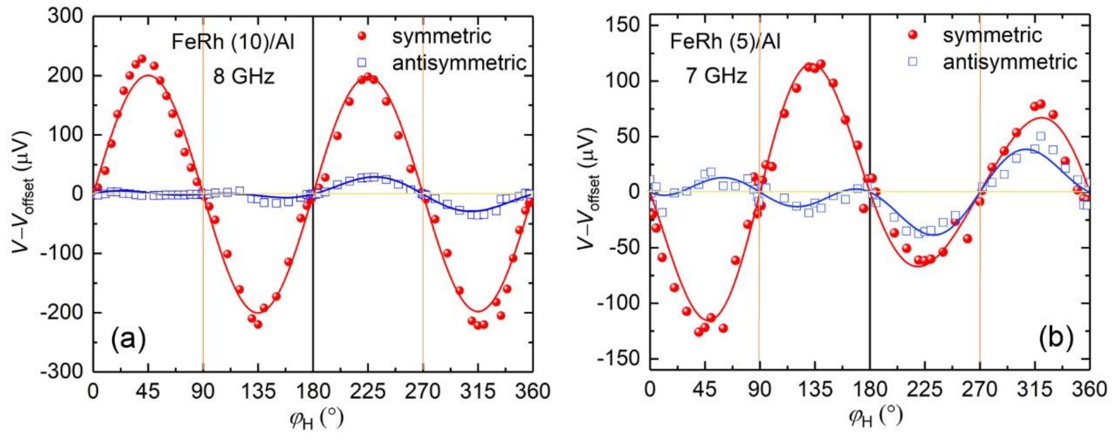

Supplementary Figure 4. Symmetric (red solid circles) and antisymmetric (blue open squares) d.c. voltages as a function of angle φ_H for (a) FeRh (10)/Al at 8 GHz, (b) FeRh (5)/Al at 7 GHz. The red and blue lines are fitting results for the symmetric and antisymmetric components, respectively. The error bars are smaller than the size of the symbols.

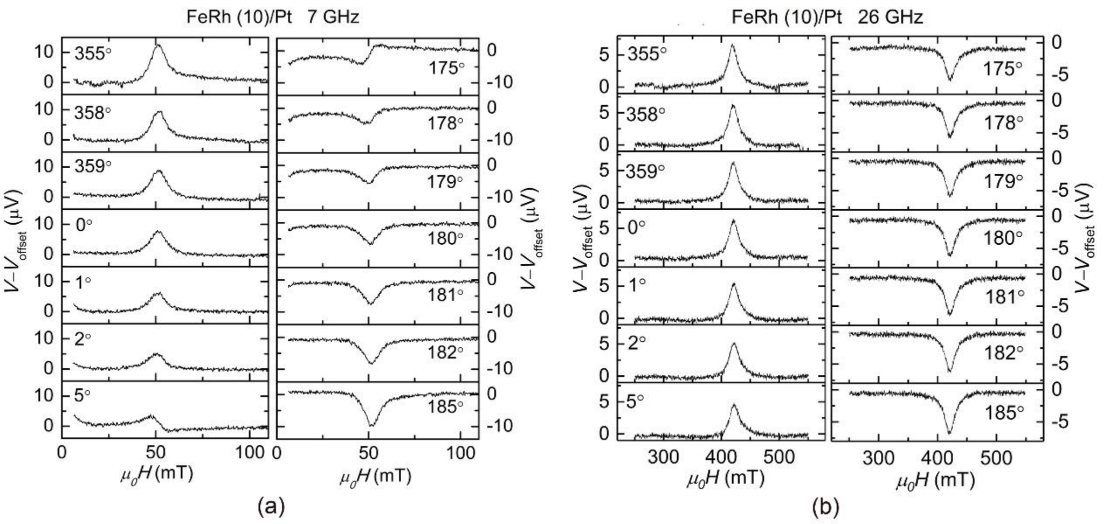

Supplementary Figure 5. d.c. voltage spectra for FeRh (10)/Pt around φ_H = 0° and 180° at 400 K and (a) 7GHz, (b) 26 GHz.

V_{ISHE} induced by spin pumping is proportional to the cross product of the spin current $\mathbf{J}_S$ (// $\mathbf{z}$) and the spin polarization vector $\boldsymbol{\sigma}$ (// $\mathbf{M}$), i.e., [3−9]

$$V_{ISHE} \sim (\mathbf{J}_S \times \boldsymbol{\sigma})_{x'} \sim [\mathrm{Im}(m_y)\mathrm{Re}(m_z) - \mathrm{Re}(m_y)\mathrm{Im}(m_z)]\cos\varphi, \quad (1)$$

where Re(m_y), Im(m_y), Re(m_z), and Im(m_z) are the real and imaginary parts of m_y and m_z. For the present measurement configuration with the direction of the dc-voltage V parallel to the inductive microwave current in FeRh $\mathbf{j}_{FeRh}(t)$, dc-voltages induced by the anisotropic magnetoresistance (AMR) of FeRh can be derived as

$$V_{\mathrm{AMR}} \sim j_{\mathrm{FeRh}} \mathrm{Re}\left(m_y\right) \sin 2\varphi. \quad (2)$$

The dynamic magnetization is connected with the dynamic magnetic susceptibility and the driving fields (for details see SI of Ref. 4, 5), and can be written as

$$\begin{pmatrix} m_y \\ m_z \end{pmatrix} = \begin{pmatrix} \chi^{\mathrm{I}} & -\mathrm{i}\chi_{\mathrm{a}}^{\mathrm{O}} \\ \mathrm{i}\chi_{\mathrm{a}}^{\mathrm{I}} & \chi^{\mathrm{O}} \end{pmatrix} \begin{pmatrix} h_{\mathrm{Oe}}^{\mathrm{Pt}} \sin\varphi \, e^{\mathrm{i}\Phi_1} \\ h^{\mathrm{O}} e^{\mathrm{i}\Phi_2} \end{pmatrix}, \quad (3)$$

where χ^{I} ($\chi_{\mathrm{a}}^{\mathrm{I}}$) is the complex diagonal (off-diagonal) dynamic magnetic susceptibility due to the in-plane excitation, χ^{O} ($\chi_{\mathrm{a}}^{\mathrm{O}}$) the complex diagonal (off-diagonal) dynamic magnetic susceptibility due to

the out-of-plane excitation, and Φ_1 (Φ_2) the phase shift between the dynamic magnetization and $h_{\mathrm{Oe}}^{\mathrm{Pt}}$ (h^{O}) [6]. Since $h_{\mathrm{Oe}}^{\mathrm{Pt}}$ is induced by the current $\mathbf{j}_{\mathrm{Pt}}(t)$ in Pt, while h^{O} is induced by the microwave current in the CPW, Φ_1 and Φ_2 are not necessarily equal, and usually $\Phi_1 \neq \Phi_2$. It should be noted that each component of the susceptibility χ has both real and imaginary parts, $\chi = \mathrm{Re}(\chi) + \mathrm{iIm}(\chi)$. Thus, from Supplementary Equation 1−3, one can derive the angular dependence of V_{ISHE} and V_{AMR} under in-plane ($h_{\mathrm{Oe}}^{\mathrm{Pt}}$) and out-of-plane ($h^{\mathrm{O}}$) excitations as

$$V_{\mathrm{ISHE}}^{\mathrm{I}} \sim \left[h_{\mathrm{Oe}}^{\mathrm{Pt}}\right]^2 \sin^2\varphi \cos\varphi \tag{4}$$

$$V_{\mathrm{AMR}}^{\mathrm{I}} \sim j_{\mathrm{FeRh}} h_{\mathrm{Oe}}^{\mathrm{Pt}} \sin 2\varphi \sin\varphi \sim j_{\mathrm{FeRh}} h_{\mathrm{Oe}}^{\mathrm{Pt}} \sin^2\varphi \cos\varphi \tag{5}$$

$$V_{\mathrm{ISHE}}^{\mathrm{O}} \sim \left[h^{\mathrm{O}}\right]^2 \cos\varphi \tag{6}$$

$$V_{\mathrm{AMR}}^{\mathrm{O}} \sim j_{\mathrm{FeRh}} h^{\mathrm{O}} \sin 2\varphi \tag{7}$$

Thus, one can obtain the following conclusions:

I) V_{ISHE} shows only a symmetric lineshape, while V_{AMR} shows both symmetric and anti-symmetric lineshapes.

II) For in-plane rotations, one cannot separate V_{ISHE} from the symmetric part of AMR, since Supplementary Equation 4 and 5 show exactly the same angular dependencies.

III) For out-of-plane excitations as used in this paper, one can separate V_{ISHE} from the symmetric part of the AMR since Supplementary Equation 6 and 7 show distinctly different angular dependence. Especially, V_{ISHE} is maximized at $\varphi = 0°$ (**M** is perpendicular to the stripe) and all other signals are zero. Thus, this geometry gives us a chance to determine V_{ISHE} accurately. We have tested the above theory experimentally and have succeeded in probing ISHE signals induced by spin pumping in a Py/Pt bi-layer [3] and at an Fe/GaAs interface [8].

Therefore, we obtain the expressions of $V_{\mathrm{a\text{-}sym}}$ and V_{sym} (Equation 1 and 2 in the main text) to fit the experimentally obtained dc-voltages. As shown in Fig. 2c and d in the main text, Equation 1 and 2 perfectly explain the experimental data, and explicit angular dependence of the dc-voltages originating

from the existence of in-plane and out-of-plane excitations. From the fitting, the magnitude of V_{ISHE} is obtained, which is the same as the value obtained in Fig. 2b of the main text, indicating again the validity of our method.

The extracted amplitudes of the symmetric voltages $V_{sym} - V_{offset}$ and antisymmetric voltages $V_{asym} - V_{offset}$ as a function of φ_H for FeRh (5)/Pt are displayed in Supplementary Figure 3, FeRh (10)/Al and FeRh (5)/Al in Supplementary Figure 4. Different from FeRh/Pt, both of $V_{sym} - V_{offset}$ and $V_{asym} - V_{offset}$ originate only from the AMR of FeRh. No ISHE voltages are detected in FeRh/Al, as evidenced by the exact zero voltage at $\varphi_H = 0°$ and 180°. This is expected due to the much smaller spin Hall angle of Al (0.0001 ~ 0.0003) in comparison to that of Pt (~ 0.1) [3].

The d.c. voltage signals of FeRh (10)/Pt at two typical frequencies (7 GHz, 26 GHz) and around $\varphi_H = 0°$ (180°) are shown in Supplementary Figure 5. At $\varphi_H = 0°$ or 180° when the in-plane magnetic fields are perpendicular to the stripe, the symmetric voltage signals show opposite polarities, which originate purely from the ISHE. When the angles deviate from $\varphi_H = 0°$ or 180°, the AMR also contributes to the d.c. voltage, which can be extracted by measuring the angular dependence of the d.c. voltage [3,4].

Supplementary Note 3: Temperature dependence of the spin pumping voltages in FeRh/Pt near the phase transition

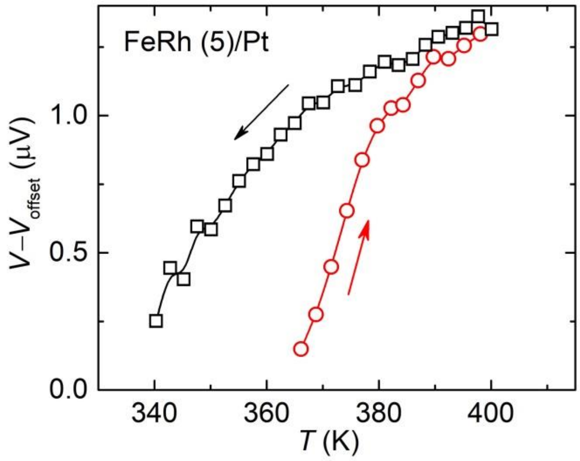

Supplementary Figure 6. ISHE voltages measured at φ_H = 0° during heating and cooling for FeRh (5)/Pt at 8 GHz.

The d.c. voltages arising from pure spin pumping as a function of T for FeRh (10)/Pt and FeRh (5)/Pt are plotted in Fig.3a and Supplementary Figure 6, respectively. According to Supplementary Equation 1 and 3, the V_{ISHE} at different temperatures for the samples can be calculated using the experimental linewidth $\mu_0\Delta H$, resonance field $\mu_0 H_R$, and effective magnetization $\mu_0 M_{eff}$ at each temperature. The calculated results are depicted as solid lines in Fig. 3a. The well-reproduced results indicate that the observed spin pumping voltage is induced by the magnetization dynamics of ferromagnetic FeRh during the phase transition. Since the magnetic properties of ferromagnetic FeRh domains remain almost unchanged during the phase transition, the decrease of the spin pumping voltage at lower T indicates that the magnetization of FeRh domains are isolated, which results in a reduced contact area with Pt and thus a reduced spin current.

Supplementary Note 4: Magnetic anisotropy analysis

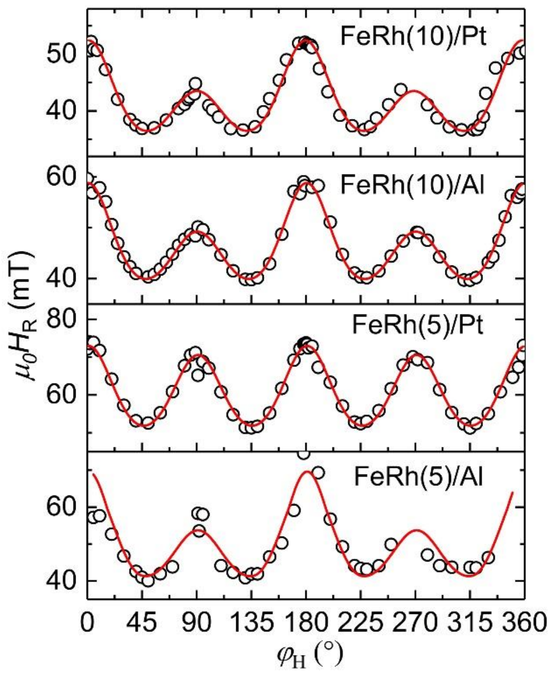

Supplementary Figure 7. In-plane angle φ_H dependent resonance field of FeRh (10)/Pt at 7 GHz, FeRh (10)/Al at 8 GHz, FeRh (5)/Pt at 8 GHz, and FeRh (5)/Al at 7 GHz. The data are fitted by solid lines to extract the magnetic anisotropy parameters of each sample. The error bars are smaller than the size of the symbols.

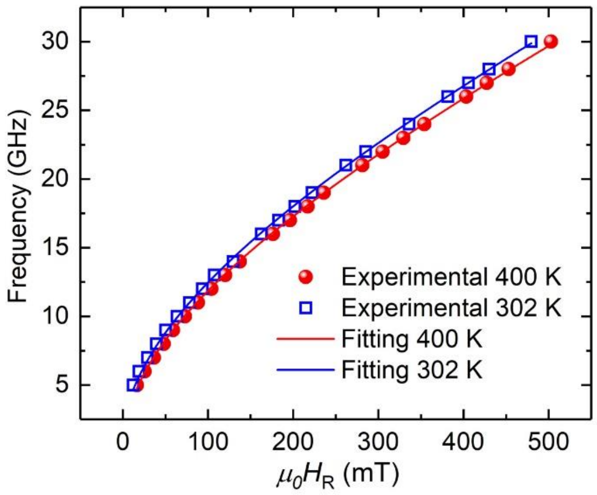

Supplementary Figure 8. Resonance field as a function of frequency measured at different temperatures for sample FeRh (10)/Pt. The data are fitted according to Supplementary Equation 8,

where $\mu_0 M_{eff}$ at different temperatures can be extracted, which has been shown in Fig. 4c in the main text.

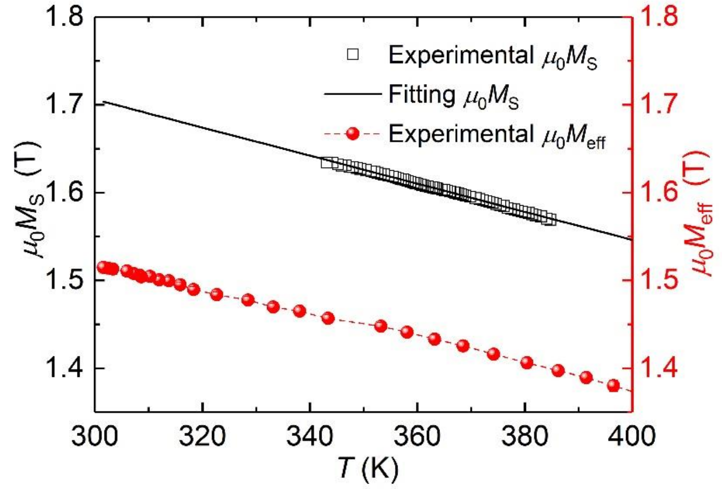

Supplementary Figure 9. Temperature dependence of the saturation magnetization $\mu_0 M_S$ and the effective magnetization $\mu_0 M_{eff}$ of FeRh (10)/Pt. The solid black line is the fitting result for $\mu_0 M_S$ as a function of T using Bloch's law.

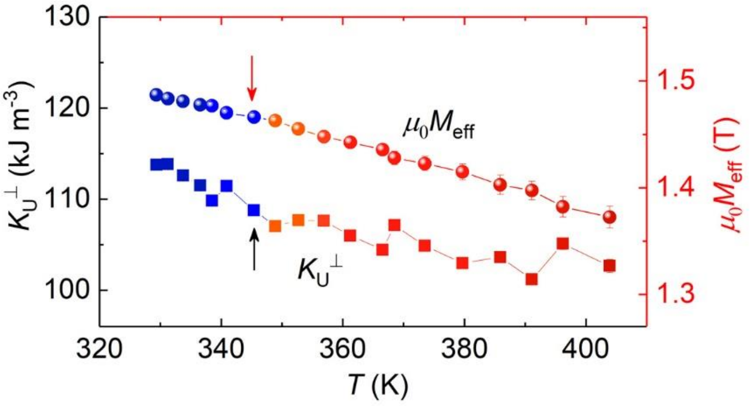

Supplementary Figure 10. Temperature dependence of $\mu_0 M_{eff}$ (spheres) and $K_U^\perp$ (squares) measured at φ_H = 45° of FeRh (10)/Pt during heating from the AFM state (lower T) to the FM state (higher T). The arrows indicate the temperature below which is the phase transition region.

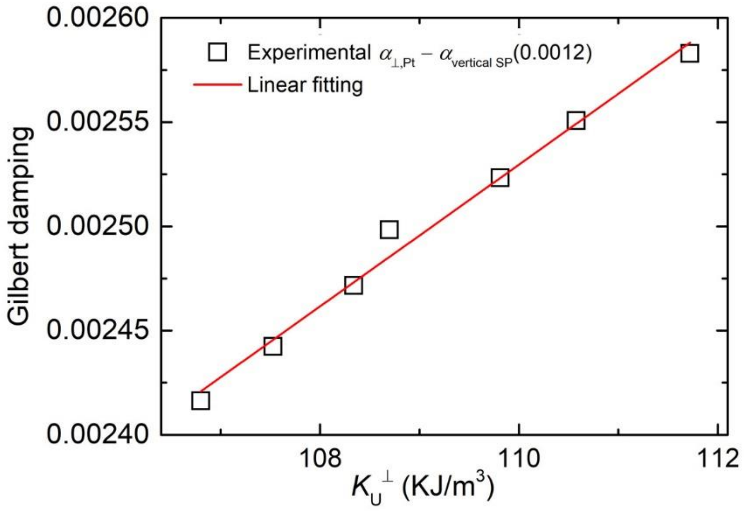

Supplementary Figure 11. Proportional relationship between intrinsic Gilbert damping $\alpha_{Gilbert}$ and $K_U^{\perp}$, where $\alpha_{Gilbert}$ linearly scales with $K_U^{\perp}$ due to the fact that both $\alpha_{Gilbert}$ and $K_U^{\perp}$ are correlated to the spin-orbit interaction. The experimental results are acquired between 350 K and 400 K where FeRh is in FM state. The error bars are smaller than the size of the symbols.

The magnetic anisotropies of the samples are investigated by analyzing the φ_H-dependent resonance field and linewidth at FMR. This data is also beneficial to identify two-magnon scattering which usually exhibits a strong in-plane anisotropy [10]. Supplementary Figure 7 summarizes the in-plane angular dependence of $\mu_0 H_R$ for each sample at 7 or 8 GHz. A cubic in-plane (biaxial) anisotropy plus a uniaxial anisotropy are observed for all samples. Specifically, FeRh [010] (φ_H = 45°) and FeRh [$\bar{1}$00] (φ_H = 135°) are easy axis, while FeRh [110] (φ_H = 0°) is the hard axis. The φ_H dependent H_R can be fitted by the resonance condition

$$(\frac{\omega}{\gamma})^2 = {\mu_0}^2 H_1 H_2 \qquad (8)$$

where $\omega = 2\pi f$ is the angular frequency, $\gamma = g\mu_B/\hbar$ is the gyromagnetic ratio, g is the spectroscopic splitting factor, μ_B is the Bohr magneton, $\hbar$ is the Dirac constant. Here $H_1 = H_R\cos(\varphi_M - \varphi_H) + \mu_0 M_{eff} + H_B(3 - \cos4\varphi_M)/4 - H_U\sin^2\varphi_M$, and $H_2 = H_R\cos(\varphi_M - \varphi_H) - H_B\cos4\varphi_M - H_U\cos2\varphi_M$ [8]. By fitting the φ_H dependence of H_R at the corresponding frequencies, the magnitude of the effective magnetization

$\mu_0 M_{eff}$, the biaxial magnetic anisotropy field $\mu_0 H_B$, and the in-plane uniaxial magnetic anisotropy field $\mu_0 H_U$ can be obtained.

Supplementary Figure 8 shows the frequency dependent resonance field $\mu_0 H_R$ of FeRh (10)/Pt at two typical temperatures, i.e., 400 K (FM state) and 302 K (phase transition). Supplementary Figure 9 summarizes the T dependence of $\mu_0 M_{eff}$ and $\mu_0 M_S$, where the experimental $\mu_0 M_S$ in the FM state are determined via SQUID measurements. At the phase transition, $M_S(T)$ can be extrapolated from Bloch's law which applies for T significantly below the Curie temperature T_C of FeRh, reading as $M_S(T) = M_S(0\ K)(1 - \beta(T/T_C)^{3/2})$ [11]. With $M_S(0\ K) = 1.98$ T, $T_C = 650$ K and critical exponent $\beta = 0.45$, the fitting results are shown as the black line in Supplementary Figure 9. Accordingly, the increase of $\mu_0 M_{eff}$ during cooling is simply due to the change of saturation magnetization $\mu_0 M_S$ of FM FeRh, which increases with decreasing temperature below T_C. Thus the T dependence of out-of-plane uniaxial anisotropy constant $K_U^{\perp}$ can be calculated according to $K_U^{\perp} = \frac{1}{2} M_S(\mu_0 M_S - \mu_0 M_{eff})$, as displayed in Fig. 4c of the main text. In addition, Supplementary Figure 10 shows the temperature dependence of $\mu_0 M_{eff}$ and $K_U^{\perp}$ during heating from the phase transition region to the FM state. Below 340 K, no abrupt change of $\mu_0 M_{eff}$ and $K_U^{\perp}$ is observed, indicating that the magnetic anisotropy of the ferromagnetic domains during their nucleation remains unchanged, consistent with the cooling procedure.

As it is known that the intrinsic Gilbert damping $\alpha_{Gilbert}$ scales linearly with $K_U^{\perp}$ due to the fact that both $\alpha_{Gilbert}$ and $K_U^{\perp}$ are second-order effects of the spin-orbit interaction perturbing the d-electron band structure. In our case, when T is in the range of 350~400 K during cooling, the whole FeRh film is in the FM state where $\alpha_{\perp,Pt} = \alpha_{Gilbert} + \alpha_{Vertical\ SP}$. Thus, one can extrapolate the magnitude of $\alpha_{Gilbert}$ during the phase transition, by using the linear relationship between $K_U^{\perp}(T)$ and $\alpha_{Gilbert}$ in the T range of 350 ~ 400 K, as shown in Supplementary Figure 11. The data shows that $\alpha_{Gilbert}$ scales linearly with $K_U^{\perp}$ with a slope of $3.40(\pm\ 0.17)\times 10^{-8}$ m^3 kJ^{-1}. And the extrapolated $\alpha_{Gilbert}$ in the whole temperature range including the phase transition region is shown in Fig. 6d of the main text.

Supplementary Note 5: Vertical spin pumping in AFM-FeRh/Py bilayers

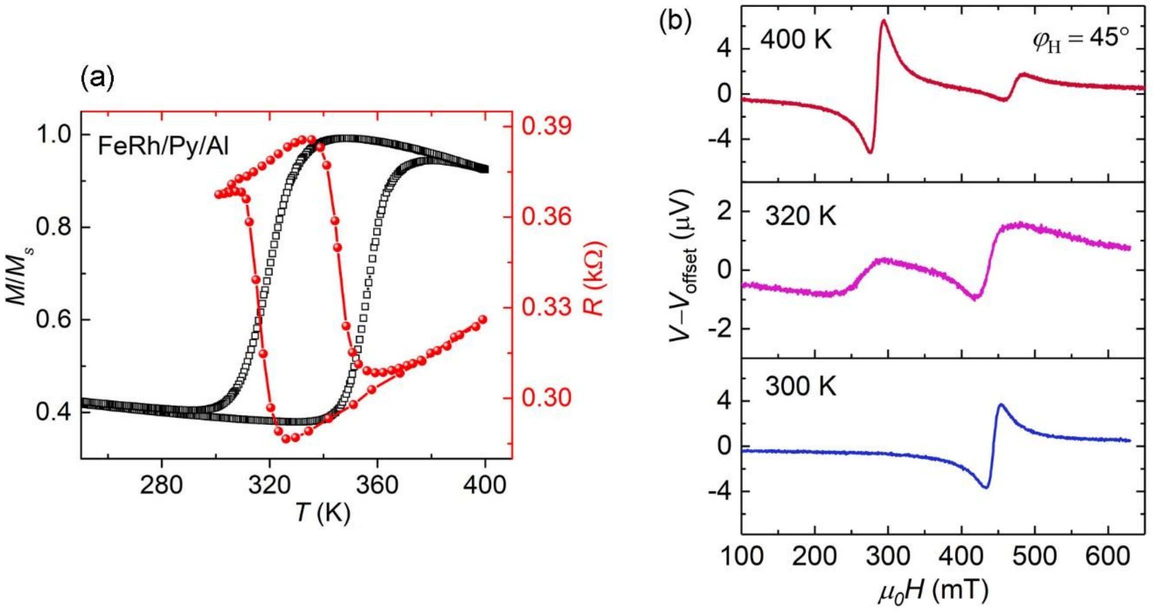

Supplementary Figure 12. (a) Temperature dependent normalized magnetization (black squares) and four-point resistance (red spheres) of FeRh/Py/Al. (b) d.c. voltage signals obtained at 22 GHz and φ_{H} = 45°.

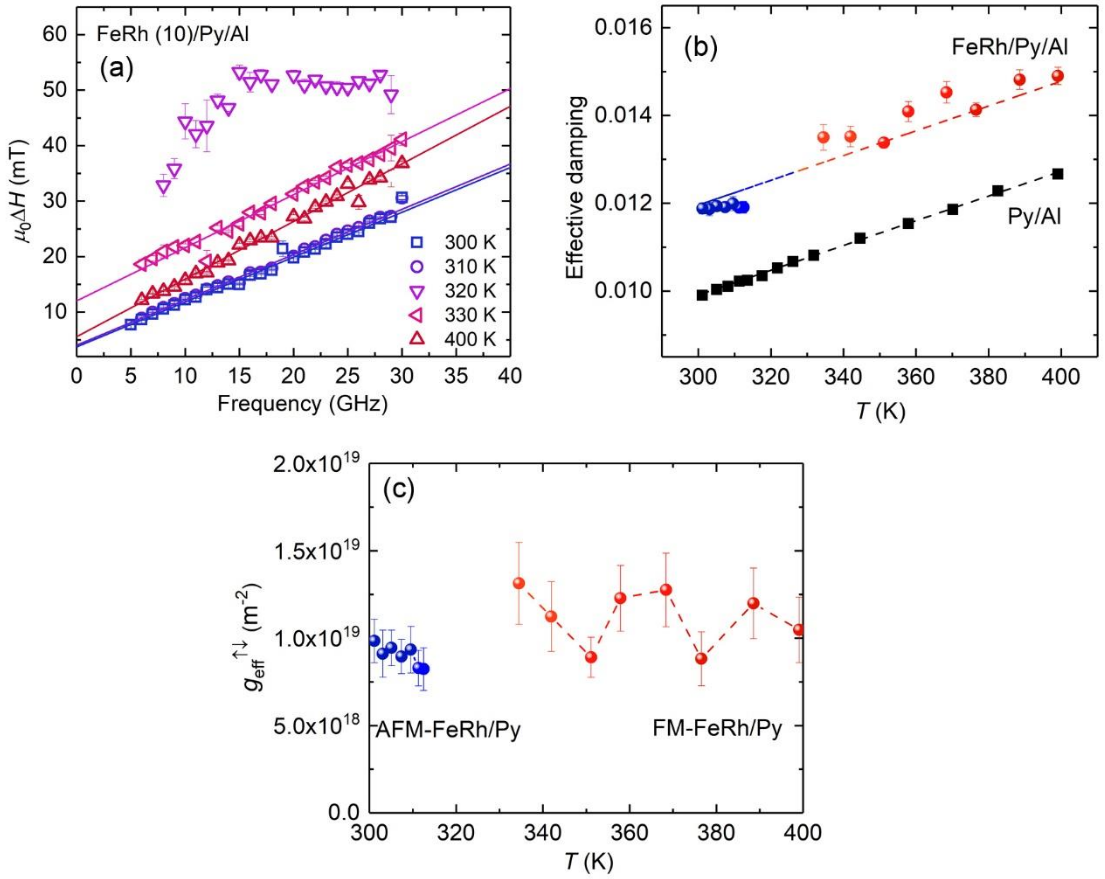

Supplementary Figure 13. (a) Frequency dependence of $\mu_0\Delta H$ at typical temperatures of FeRh/Py/Al. (b) Corresponding effective damping of FeRh/Py/Al and Py/Al as a function of temperature. (c) Temperature dependence of the spin mixing conductance at the interface of FeRh/Py. The data are acquired at φ_H = 45°. The error bars of damping are standard deviations obtained from the linear fitting of the linewidth, which also induce error bars for estimation of $g_{\mathrm{eff}}^{\uparrow\downarrow}$.

Due to the strong spin-orbit coupling arising from the 4*d* electrons of Rh in AFM-FeRh, FeRh can act as a spin sink similar to heavy normal metals. Here, we also investigate vertical spin pumping in FeRh (10)/Py (15)/Al. The temperature dependent magnetization and resistance loops in Supplementary Figure 12a display the phase transition in FeRh/Py. Supplementary Figure 12b shows the dc-voltage signals measured at 22 GHz at different temperatures. When FeRh is in the FM state at 400 K, two peaks are detected at the resonance field of FM-FeRh (lower H_R) and Py (higer H_R), respectively. With decreasing the temperature, when FeRh transforms into the AFM state, only the resonance peak from Py is detectable.

By analyzing the frequency dependent $\mu_0\Delta H$ of Py at different temperatures (Supplementary Figure 13a), the effective damping of Py during the phase transition of FeRh is obtained. Note that *f*-$\mu_0\Delta H$ has nonlinear behavior during the phase transition (around 320 K), probably because of linewidth broadening related to inhomogeneities at the FeRh/Py interface or/and two-magnon scattering. Thus, it is hard to extract the correct damping value of Py during the phase transition. Supplementary Figure 13b compares the temperature dependence of damping in FeRh/Py/Al and Py/Al. An enhancement of damping in FeRh/Py/Al ($\Delta\alpha_{SP}$ ~ 0.002) is observed, due to spin pumping from Py into FeRh. Accordingly, the effective spin mixing conductance at the interface of FeRh and Py can be estimated (Supplementary Figure 13c) by $g_{\mathrm{eff,FeRh/Py}}^{\uparrow\downarrow} = \frac{4\pi M_S d_{\mathrm{Py}}}{\gamma\hbar}\left(\alpha_{\mathrm{FeRh/Py}} - \alpha_{\mathrm{Py}}\right)$ [12]. Note that both of FM-FeRh and AFM-FeRh can act as spin sinks, and in both cases the spin mixing conductance is similar. The magnitude of $g_{\mathrm{eff}}^{\uparrow\downarrow}$ is almost temperature independent in the investigated temperature range.

Furthermore, the spin Hall angle θ_{SHE} of FeRh can be quantified by

$$V_{\mathrm{ISHE}} = RI_C = R\frac{2\mathrm{e}}{\hbar}\theta_{SHE}J_S w\lambda \tanh\frac{d}{2\lambda}\,, \quad (9)$$

where R is the resistance of the FeRh/Py bi-layer, I_C the charge current induced by the inverse spin Hall effect, e the electronic charge, $\hbar$ the Dirac constant, w the width of the stripe, λ the spin diffusion length of FeRh, d the thickness of FeRh, and J_S the magnitude of spin current at the interface. Instead of θ_{SHE}, we use the product of θ_{SHE} and λ, $\theta_{SHE}\lambda$ in unit of nm, to determine the spin-to-charge efficiency [13]. Based on the condition that λ of FeRh is assumed to be much smaller than d, which holds also for other AFMs [14], Supplementary Equation 9 can be rewritten as:

$$I_{\mathrm{C}} = (\theta_{\mathrm{SHE}}\lambda)\frac{2\mathrm{e}}{\hbar}J_S w. \quad (10)$$

The magnitude of J_S can be determined by

$$J_S = \frac{\hbar\omega}{4\pi} g_{\mathrm{eff}}^{\uparrow\downarrow} \frac{1}{M^2}\left[\mathrm{Im}(m_y)\mathrm{Re}(m_z) - \mathrm{Im}(m_z)\mathrm{Re}(m_y)\right], \quad (11)$$

where ω is the angular frequency ($\omega = 2\pi f$), Re(m_y) (Re(m_z)) the real part of the dynamic magnetization m_y (m_z), and Im(m_y) (Im(m_z)) the imaginary part of the dynamic magnetization m_y (m_z). As shown in Fig. 5d of the maintext, an approximately two times larger $\theta_{SHE}\lambda$ is found in the AFM phase, indicating that AFM-FeRh can be a good spin sink. In short, the observed vertical spin pumping in AFM-FeRh/Py confirms the important role of the AFM-FeRh matrix as a spin sink, supporting the main idea of lateral spin pumping from FM-FeRh into AFM-FeRh of this work.

Supplementary Note 6: In-plane anisotropic linewidth and two-magnon scattering

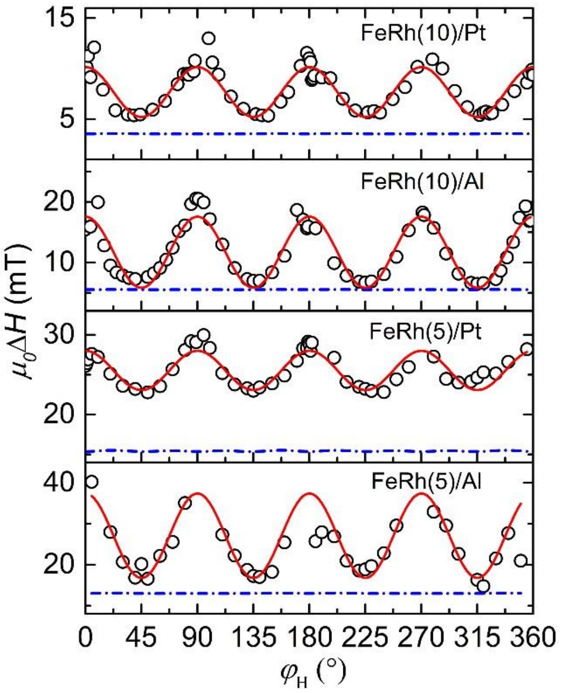

Supplementary Figure 14. In-plane angle φ_H dependent linewidth of FeRh (10)/Pt at 7 GHz, FeRh (10)/Al at 8 GHz, FeRh (5)/Pt at 8 GHz, and FeRh (5)/Al at 7 GHz. The solid red lines fit to the data according to two-magnon scattering, and the dashed dotted lines are the calculated intrinsic linewidth plus inhomogeneity broadening of linewidth. The error bars are smaller than the size of the symbols.

Supplementary Figure 14 shows the angular dependence of the linewidth for the four different FeRh samples, which exhibit a pronounced four-fold symmetry. This symmetry is expected by neither intrinsic damping nor inhomogeneous broadening, and is the signature of two-magnon scattering, which has a $\cos^2(2\varphi_H)$ dependence. The anisotropic $\mu_0\Delta H$ is well fitted by the combination of two-magnon scattering and intrinsic damping as shown by red solid lines. Similar phenomena have also been reported and theoretically studied in other ferromagnetic films such as Fe [10,15,16]. Although two-magnon scattering is evidenced to contribute to the total $\mu_0\Delta H$, the magnitude of the angular dependent two-magnon scattering contribution during cooling does not increase a lot compared to the dramatic enhancement of $\mu_0\Delta H$ shown in Fig. 3b of the main text. Thus two-magnon scattering is not the dominating origin for the enhanced $\mu_0\Delta H$.

Supplementary Note 7: Analysis of frequency-dependent linewidth and effective damping

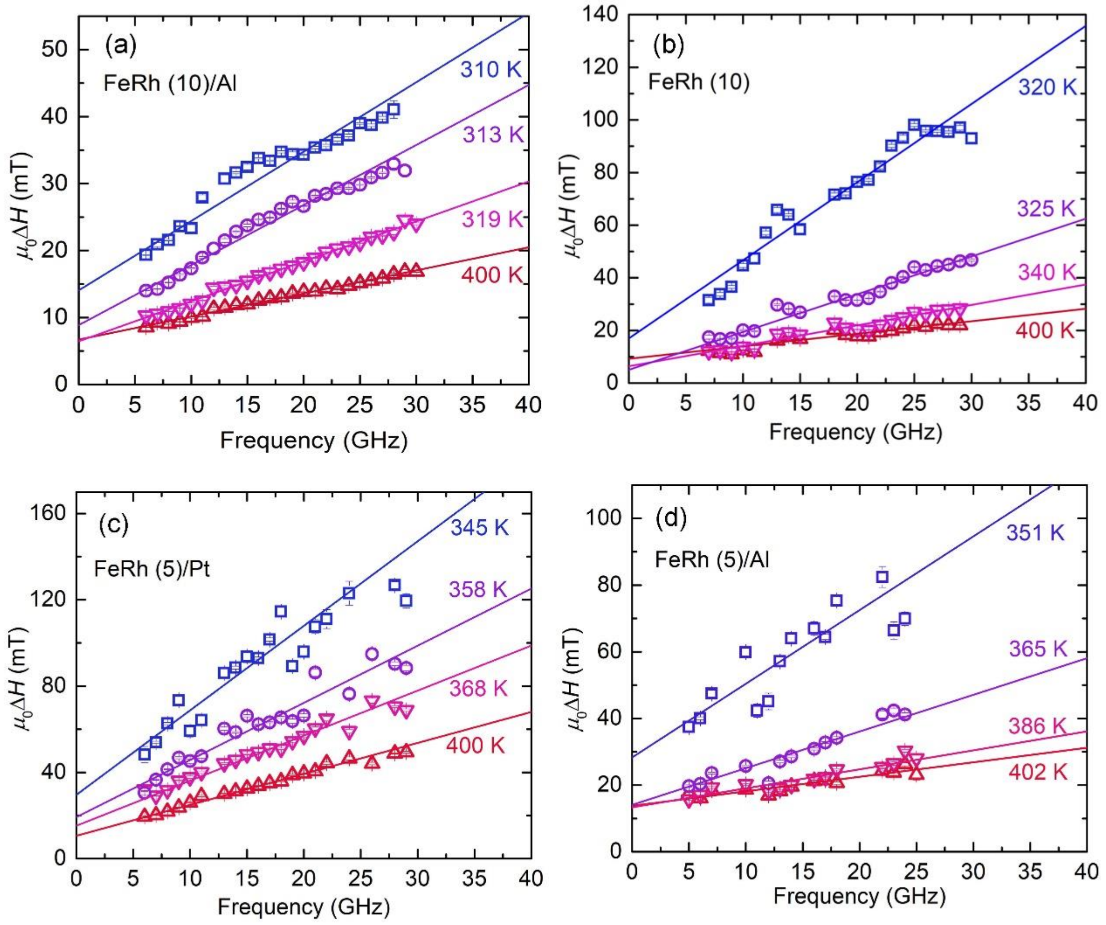

Supplementary Figure 15. Frequency dependence of $\mu_0\Delta H$ at typical temperatures of samples (a) FeRh (10)/Al, (b) FeRh (10), (c) FeRh (5)/Pt, (d) FeRh (5)/Al. Here $\mu_0\Delta H$ values are extracted from the in-plane spin pumping measurements at φ_H = 45° during cooling from FM state to AFM state. The error bars are standard deviations obtained from the fit of d.c. voltage loops.

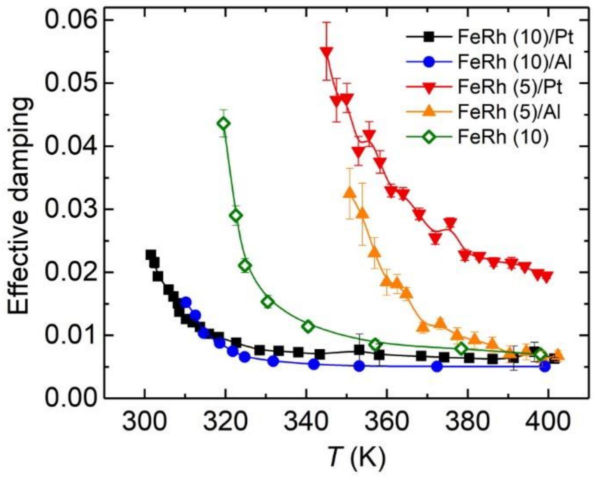

Supplementary Figure 16. The effective damping for five different samples as a function of T during cooling. The error bars are obtained from the linear fitting of the linewidth.

In addition to the linear fitting of f-dependent $\mu_0\Delta H$ for the sample FeRh (10)/Pt, we also show results for FeRh (10)/Al, FeRh (10), FeRh (5)/Pt, and FeRh (5)/Al in Supplementary Figure 15. Although two-magnon scattering could lead to nonlinear f dependence [10,15,16], it is hard to distinguish in our experiments due to the scatter of the data. Thus, linear fitting of $\mu_0\Delta H$ as a function of f is adopted to allow a comparison between different T. In general, a larger value of zero-frequency inhomogeneous linewidth $\mu_0\Delta H_0$ is observed at lower T, indicating the increase of inhomogeneity when FeRh goes into the phase transition region. This is probably because some FM domains nucleate and grow in the AFM matrix during the phase transition, bringing about inhomogeneous crystalline structure. According to the fitting Equation 4 in the main text, the effective damping for all of the five samples can be obtained, as summarized in Supplementary Figure 16. It is noticed that the damping of FeRh (10) without capping layers is larger than FeRh capped either with Pt or Al. This is as expected since the surface of FeRh is easily oxidized in air, leading to a mixed state of FM- and AFM-FeRh. Meanwhile, the inhomogeneity and possible defects of the surface layer due to the degradation in air could also increase the linewidth and damping. Nevertheless, for different FeRh samples with different transition temperatures, α_{eff} increases dramatically in the respective phase transition regions. As discussed in the main text, the enhancement of α_{eff} is caused by the lateral spin pumping between FM and AFM FeRh.

Supplementary Note 8: Out-of-plane film-FMR measurements on FeRh/(Pt, Al)

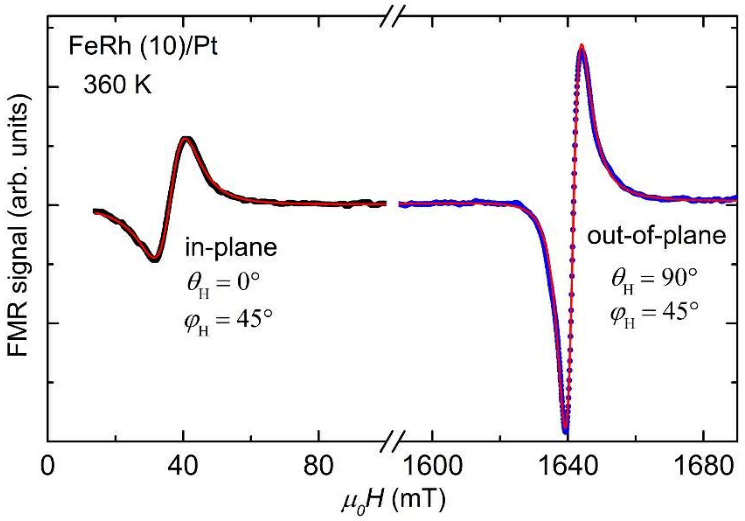

Supplementary Figure 17. Typical FMR spectra at θ_H = 0° (in-plane) and 90° (out-of-plane) for sample FeRh (10)/Pt at 360 K when FeRh is in FM state.

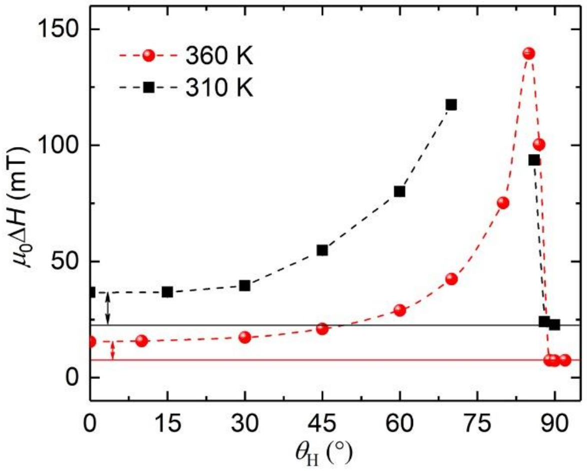

Supplementary Figure 18. Out-of-plane angle θ_H dependent linewidth at 360 K and 310 K in full film-FMR measurements for FeRh (10)/Pt measured at 7 GHz.

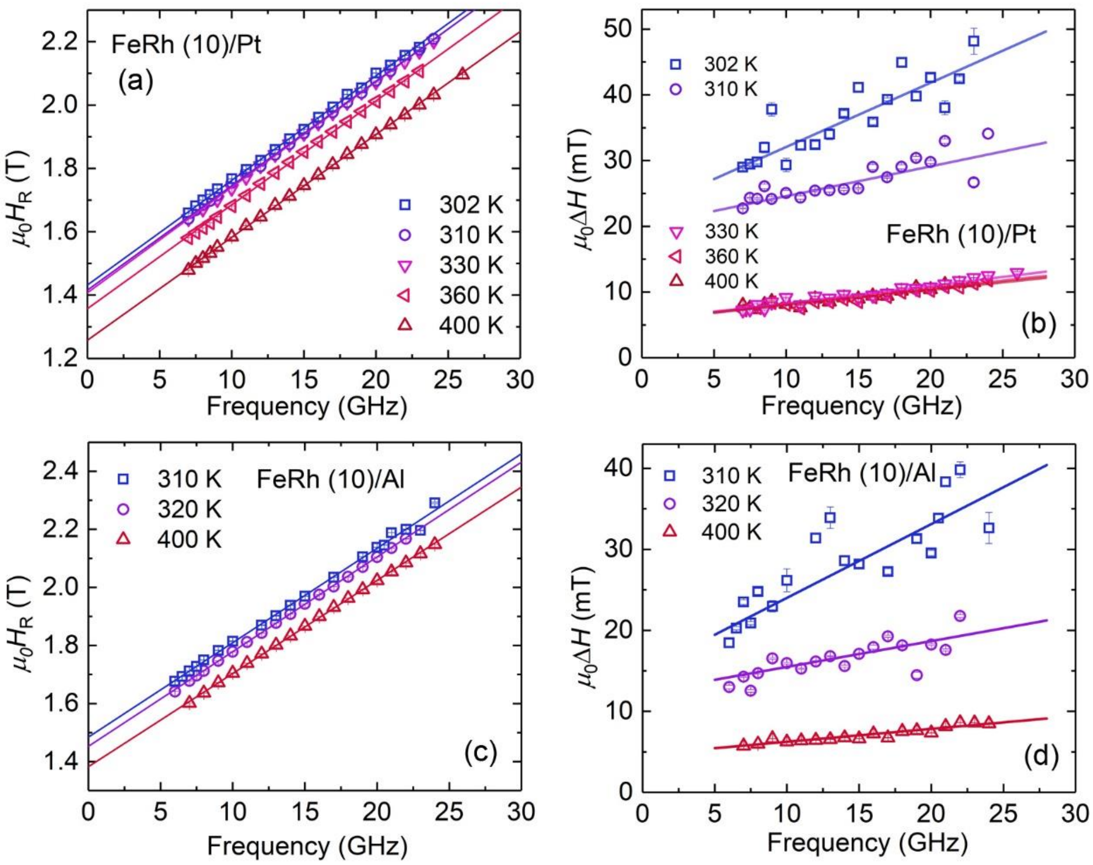

Supplementary Figure 19. (a,b) $\mu_0 H_R$ and $\mu_0 \Delta H$ as a function of frequency measured at $\theta_H = 0°$, $\varphi_H = 45°$ (out-of-plane) measured at different temperatures (during cooling) for FeRh (10)/Pt thin film. (c,d) The same for FeRh (10)/Al, where 400 K corresponds to FM state and 310 K corresponds to phase transition. The error bars are standard deviations obtained from the fit of the out-of-plane FMR loops.

In this part, we show the standard full film-FMR results of the samples FeRh (10)/Pt and FeRh (10)/Al. Supplementary Figure 17 compares the typical FMR spectra at 7 GHz when external magnetic fields are in-plane ($\theta_H = 90°$, $\varphi_H = 45°$) and out-of-plane ($\theta_H = 0°$, $\varphi_H = 45°$). The FMR spectra measured by lock-in technique are fitted by a combination of derivative symmetric and anti-symmetric Lorentzians with respect to the external magnetic field H [17],

$$L_{\mathrm{FMR}} = \frac{A_{\mathrm{sym}}[\left(\frac{\Delta H}{2}\right)^2 - (H-H_R)^2]}{[(H-H_{\mathrm{R}})^2+\left(\frac{\Delta H}{2}\right)^2]^2} + \frac{2A_{\mathrm{asym}}\frac{\Delta H}{2}(H-H_{\mathrm{R}})}{[(H-H_{\mathrm{R}})^2+\left(\frac{\Delta H}{2}\right)^2]^2} \tag{12}$$

where A_{sym} and A_{asym} are the magnitudes of symmetric part and antisymmetric part respectively. In order to clarify the contribution of two-magnon scattering, the sample is measured during out-of-plane rotation from θ_H = 0° to θ_H = 90° where φ_H is fixed to 45°. As shown in Supplementary Figure 18, the linewidth for the out-of-plane direction (θ_H = 90°) is smaller than for the in-plane direction (θ_H = 0°), which clearly proves the contribution of two-magnon scattering in the in-plane configuration [10,15,16]. The magnitude of two-magnon scattering is predicted to be enhanced during the phase transition, through comparing the length of the two arrows for the 310 K case and the 360 K case shown close to 0° in Supplementary Figure 18. This result is consistent with the increased α_{2M} (T) related to two-magnon scattering when decreasing the temperature, as discussed in Fig. 6d of the main text.

Supplementary Figure 19 displays the frequency dependent resonance field $\mu_0 H_R$ and linewidth $\mu_0 \Delta H$ for the samples FeRh (10)/Pt and FeRh (10)/Al. In the out-of-plane configuration, the resonance condition simply reads

$$\frac{2\pi f}{g\mu_B/\hbar} = \mu_0(H_R - M_{eff}) \qquad (13)$$

where $\mu_0 H_R$ has a linear relationship with f. By fitting the data using Supplementary Equation 13, $\mu_0 M_{eff}$ can be directly read as the intercept at 0 GHz. The increase of $\mu_0 M_{eff}$ at lower T is consistent with that measured in the in-plane configuration (Fig. 4c of the main text), regardless of the slightly larger value. g is estimated to be in the range between 2.14 and 2.19 for both samples, without clear temperature dependence [18,19]. In addition, through linear fitting of f-dependent $\mu_0 \Delta H$ in Supplementary Figure 19b and d, one can find that the slope increases with decreasing temperatures, indicating enhanced damping during the phase transition. As discussed in the main text for the out-of-plane configuration, the damping $\alpha_{\perp,Pt}(T)$ for FeRh (10)/Pt increases from 0.0035 to 0.0147 by a factor of 4 when cooling from the FM to the FM-AFM co-existence state. For sample FeRh (10)/Al, an increase of damping $\alpha_{\perp,Al}(T)$ from 0.0023 (400 K) to 0.0134 (310 K) is also observed. The values are smaller for FeRh(10)/Al compared to that of FeRh(10)/Pt, verifying the vertical spin pumping between FeRh and Pt. Note that no full film-FMR signal is detected when t is reduced to 5 nm. The high sensitivity of spin pumping measurements presents a great advantage beyond standard full film-FMR

for investigating spin dynamics. Accordingly, the enhanced damping $\alpha_{\mathrm{Vertical\ SP}}$ by vertical spin pumping from FeRh into Pt can be estimated, through comparing $\alpha_{\perp,\mathrm{Pt}}(T)$ and $\alpha_{\perp,\mathrm{Al}}(T)$. Due to the different phase transition temperatures for FeRh/Pt and FeRh/Al, $\alpha_{\mathrm{Vertical\ SP}}$ is generally estimated for FM states [0.0035 ($\alpha_{\perp,\mathrm{Pt}}$(400 K)) – 0.0023 ($\alpha_{\perp,\mathrm{Al}}$(400 K)) = 0.0012] and during the phase transition [0.0147 ($\alpha_{\perp,\mathrm{Pt}}$(302 K)) – 0.0134 ($\alpha_{\perp,\mathrm{Al}}$(310 K)) = 0.0013]. It is then assumed that $\alpha_{\mathrm{Vertical\ SP}}$ stays constant during the phase transition, compared to the greatly enhanced total effective damping α_{eff} and $\alpha_{\mathrm{Lateral\ SP}}$. This is expected since the magnetization in isolated ferromagnetic FeRh pillars and at the interface between FeRh and Pt are basically unchanged during the phase transition, where only the contact areas are reduced. From another point of view, the dramatic increase of effective damping for FeRh/Al (5 nm or 10 nm) during the phase transition directly excludes the contribution from vertical spin pumping. Moreover, since two-magnon scattering is turned off when FeRh/Al is measured by out-of-plane FMR, the contributions to the obtained $\alpha_{\perp,\mathrm{Al}}(T)$ only stem from intrinsic Gilbert damping and lateral spin pumping: $\alpha_{\perp,\mathrm{Al}}(T) = \alpha_{\mathrm{Gilbert,Al}}(T) + \alpha_{\mathrm{Lateral\ SP,Al}}(T)$. As expected, the dramatically increased damping during the phase transition of FeRh/Al can be mainly attributed to lateral spin pumping and its value ($\alpha_{\mathrm{Lateral\ SP,Al,310K}}$ = 0.0103) is quite close to the value ($\alpha_{\mathrm{Lateral\ SP,Pt,302K}}$ = 0.0109) for FeRh/Pt. This further verifies the observation of lateral spin pumping during the phase transition of FeRh, which is independent of the capping material (Pt or Al).

Supplementary References

[1] Lewis, L. H., Marrows, C. H. & Langridge, S., Coupled magnetic, structural, and electronic phase transitions in FeRh. J. Phys. D: Appl. Phys. **49**, 323002 (2016).

[2] Uhlíř, V., Arregi, J. A. & Fullerton, E. E. Colossal magnetic phase transition asymmetry in mesoscale FeRh strpes. Nat. Commun. **7**, 13113 (2016).

[3] Obstbaum, M., Härtinger, M., Bauer, H. G., Meier, T., Swientek, F., Back, C. H. & Woltersdorf, G. Inverse spin Hall effect in $Ni_{81}Fe_{19}$/normal-metal bilayers. Phys. Rev. B **89**, 060407(R) (2014).

[4] Chen, L., Matsukura, F. & Ohno, H. Direct-current voltages in (Ga,Mn)As structures induced by ferromagnetic resonance. Nat. Commun. **4**, 2055 (2013).

[5] Chen, L. et al. Emergence of anisotropic Gilbert damping in ultrathin Fe layers on GaAs(001). Nat. Phys. **14**, 490–494 (2018).

[6] Harder, M., Cao, Z. X., Gui, Y. S., Fan, X. L. & Hu, C. -M. Analysis of the line shape of electrically detected ferromagnetic resonance. Phys. Rev. B **84**, 054423 (2011).

[7] Gilbert, T. L. A phenomenological theory of damping in ferromagnetic materials. IEEE Trans. Magn. **40**, 3443–3449 (2004).

[8] Chen, L., Decker, M., Kronseder, M., Islinger, R., Gmitra, M., Schuh, D., Bougeard, D., Fabian, J., Weiss, D. & Back, C.H. Robust spin-orbit torque and spin-galvanic effect at the Fe/GaAs (001) interface at room temperature. Nat. Commun. **7**, 13802 (2016).

[9] Ando, K., Kajiwara, Y., Takahashi, S., Maekawa, S., Takemoto, K., Takatsu, M. & Saitoh, E. Angular dependence of inverse spin-Hall effect induced by spin pumping investigated in a $Ni_{81}Fe_{19}$/Pt thin film. Phys. Rev. B **78**, 014413 (2008).

[10] Zakeri, Kh. et al. Spin dynamics in ferromagnets: Gilbert damping and two-magnon scattering. Phys. Rev. B **76**, 104416 (2007).

[11] Zhukov, A. Novel Functional Magnetic Materials - Fundamentals and Applications. Springer International Publishing Switzerland, 2016.

[12] Taserkovnyak, Y., Brataas, A., Bauer, G. E. & Halperin, B. I. Nonlocal magnetization dynamics in ferromagnetic heterostructures. Rev. Mod. Phys. **77**, 1375–1421 (2005).

[13] Rojas-Sánchez, J.-C. et al. Spin pumping and inverse spin Hall effect in platinum: the essential role of spin-memory loss at metallic interfaces. Phys. Rev. Lett. **112**, 106602 (2014).

[14] Zhang, W., Jungfleisch, M. B., Jiang, W., Pearson, J. E., Hoffmann, A., Freimuth, F. & Mokrousov, Y. Spin Hall effects in metallic antiferromagnets. Phys. Rev. Lett. **113**, 196602 (2014).

[15] Woltersdorf, G. & Heinrich, B. Two-magnon scattering in a self-assembled nanoscale network of misfit dislocations. Phys. Rev. B **69**, 184417 (2014).

[16] Lenz, K., Wende, H., Kuch, W., Baberschke, K., Nagy, K. & Jánossy, A. Two-magnon scattering and viscous Gilbert damping in ultrathin ferromagnets. Phys. Rev. B **73**, 144424 (2006).

[17] Celinski, Z., Urquhart, K. & Heinrich, B. Using ferromagnetic resonance to measure the magnetic moments of ultrathin films. Journal of Magnetism and Magnetic Materials **166**, 6–26 (1997).

[18] Stamm, C. et al. Antiferromagnetic-ferromagnetic phase transition in FeRh probed by x-ray magnetic circular dichroism. Phys. Rev. B **77**, 184401 (2008).

[19] Hrabec, A., Gonçalves, F. J. T., Spencer, C. S., Arenholz, E., N'Diaye, A. T., Stamps, R. L. & Marrows, C. H. Spin-orbit interaction enhancement in permalloy thin films by Pt doping. Phys. Rev. B **93**, 014432 (2016).
